# Consumer willingness to pay for plant-based foods produced using microbial applications to replace synthetic chemical inputs

**DOI:** 10.1371/journal.pone.0260488

**Published:** 2021-12-07

**Authors:** Beshir M. Ali, Frederic Ang, H. J. van der Fels-Klerx

**Affiliations:** Business Economics Group, Wageningen University & Research, Wageningen, the Netherlands; Groupe ESC Dijon Bourgogne, FRANCE

## Abstract

Analysis of consumer preferences and willingness-to-pay (WTP) for sustainable foods produced using new agri-food technologies is required to enhance the uptake of innovations that accelerate the transition towards sustainable food systems. Consumers’ willingness to buy new food products, with no or limited consumption experience, mainly depends on their food choice motivational orientations (promotion- vs prevention-orientation). The objective of this study was to elicit consumers’ WTP for foods that are produced with microbial applications during the plant production phase with the aim to reduce the use of synthetic chemicals in crop farming, as well as to understand the associations of food choice motives, personal and socio-demographic factors with the WTP. We used contingent valuation to elicit consumers’ WTP for three food products (wheat bread, consumer potatoes and tomato sauce) through online surveys. Data were collected from 291 consumers, primarily from Italy, Germany and the Netherlands. Descriptive statistics, latent variable modelling and logistic regression were used to analysis data. Results show that more than two-third of the respondents are willing to pay premiums of at least 0.11 euro per kg of food products for reductions in synthetic chemical use by at least 50% due to microbial applications. The amount of WTP increases with the level of reductions in synthetic chemical use. The majority of the respondents are promotion-oriented consumers in relation to their food involvement, and are more likely to pay premiums for the sustainably produced food products. Environmentally concerned consumers are also more likely to pay premiums, whereas health concerned consumers are not. This study contributes to understanding of consumers’ attitude and perceived health risks towards foods obtained using microbial applications, and the heterogeneity of their preferences. Results provide insights for identifying potential buyers of foods produced using microbial applications, and to set prices according to the levels of consumers’ WTP.

## Introduction

Global food systems are currently characterised by unsustainable production and consumption practices [1, 2]. Agriculture is currently responsible for about 23% of human-induced greenhouse gases (GHGs) emissions [3], more than 50% of water withdrawals [4], and 75% of biodiversity losses [5]. In spite of these environmental impacts, a 50% increase in agricultural output is required to feed the estimated 9.7 billion world population by 2050, which exerts additional pressure on the carrying capacity of the environment [5]. Adoption of sustainable practices in production (e.g. circular farming) and consumption decisions (e.g. consumption shift from animal- to plant-based food sources or within animal/plant food sources from high impact to low impact products) is required to materialise the transition towards sustainable and healthy food systems. Springmann et al. [6] reported that Western countries should reduce beef consumption by 90% and increase the consumption of lentils and beans by 400% to achieve a sustainable and healthy global food system.

The adoption of novel agri-food technologies by value chain actors has been improving the safety, sustainability and nutritional value of food [7]. Unfortunately, consumers’ attitude towards these technologies is not always positive; as noted by Siegrist and Hartmann [7] that contrary to the views of experts, “many consumers perceive the use of food technologies as contradictory to healthy, nutritious and tasty food”. Investments in agri-food technologies by farmers and other value chain actors affect their competitiveness, profitability and survival. Thus, investors must make prudent adoption decisions, by thoroughly understanding consumers’ preferences and their willingness-to-pay (WTP) for sustainable and healthy food products that are produced using new agri-food technologies.

A change in consumption behaviour is required to effectively address the challenges of mitigating climate change and adapting to sustainable farming systems [2]. The willingness of consumers to buy new food products, with no or limited consumption experience, depends on their Food Choice Motives (FCMs), and personal and socio-demographic characteristics, as well as the attributes of the products [8, 9]. FCMs influence an individual’s food choices and are specifically recognized as the main drivers of purchasing decisions for new food products. FCMs consist of a wide range of factors such as price, sensory appeal, health, natural content, familiarity, convenience, mood, weight control and ethical concerns [10–12]. However, de Boer *et al*. [9, 13] identified and validated attitudinal (motivational) orientations of consumers as the main FCMs that influence one’s purchasing decisions of sustainable and healthy food products. Consumers’ food choice decisions are regulated by two distinct motivational orientations [9]: promotion/approach and prevention/avoidance. The promotion system, according to de Boer *et al*. [9] is “concerned with obtaining nurturance (e.g. nourishing food); it underlies higher-level concerns with the pleasurable presence of positive outcomes, including accomplishments, aspirations and ideals, [whereas] the prevention system is concerned with obtaining security and avoiding negative outcomes (e.g. dangerous food); it underlies higher-level concerns with safety and fulfilment of responsibilities”. According to the authors, “an individual’s momentary focus on promotion or prevention will depend on his or her personal history and circumstances induced by the situation at hand”. Furthermore, other psychological factors such as attitude (towards the use of a new technology), knowledge about the new technology, and individuals’ level of environmental and health concerns are also underlying factors for explaining consumers’ heterogeneous food preference and purchasing behaviour [14, 15]. Understanding the heterogeneity of consumers’ preference makes it possible to characterise consumer profiles and identify consumer segments, which are essential for designing marketing strategies [14].

Through its “Farm to Fork Strategy”, the European Commission (EC) aims to reduce the use of fertilisers by at least 20%, and the use and risk posed by pesticides by 50% by 2030 [16]. The use of synthetic chemicals (e.g. fertilisers, pesticides) in intensive crop farming causes environmental impacts through the release of pollutants to the air (e.g. greenhouse gases), soil (e.g. nitrogen and phosphorus) and water (e.g. nitrate and phosphate). These chemicals can also be found as residues in food products and have been linked to public health risks, such as incidences of cancer [17, 18]. Moreover, it has been shown that most European consumers are aware and worried about the impacts of synthetic chemicals on the environment (90%) and on their health (85%) [19]. In response to this growing public awareness and need for more sustainable and healthier food systems, several innovations have been developed and adopted by actors in the food value chain. Scientists are looking to apply microbial consortia to sustainably increase crop production and food quality [20]. Microbial innovations are thought to “substantially contribute to increased farm productivity, resilience to global change, profitability and sustainability, while considerably reducing chemical inputs” [21].

The use of microorganisms such as bacteria, micro-algae and fungi in crop farming and food processing has been identified as an important innovation for enhancing circular farming [22–24] and may play an essential role to the European Union’s Farm to Fork Strategy. The use of more environmentally friendly fertilizers, mostly based on plant-beneficial microbes, helps to reduce chemical use in farming and the subsequent chemical residues in the environment and food products [25]. Microbial applications can enhance the resistance of crops to biotic and abiotic stress, increase nutrient uptake, stimulate germination and plant growth, protect against diseases and soil-borne pathogens, and enhance soil quality and health [23, 24], thus reducing the need for synthetic chemical inputs in farming [21]. Microbial applications can also be used as a bioremediation for degrading pesticides in pesticide-contaminated soils [23, 26]. Furthermore, microbiome-based products can improve the quality of grains, fruits and other plant products [27], with less chemical contamination and allergens [28]. They can also improve the nutritional value of food products through increasing micronutrient and antioxidant contents [29, 30]. Micronutrients (e.g. phenols, chlorophyll and polyphenols) have a preventive role in cancer, neurodegenerative and cardiovascular disorders [31–33]. There are also several other health promoting compounds that can be obtained as a result of using microorganisms in food production [34]. In addition, soil microbiomes are positively associated with beneficial gut microbiomes [35].

In light of the foregoing discussion, the objectives of this study were: (i) to elicit consumers’ WTP for ‘sustainable and healthy’ food products that are produced with microbial applications during the plant production phase, and (ii) to estimate the effects of consumers’ FCMs, and product, personal and socio-demographic characteristics on their WTP for these food products. The research questions and the main hypotheses were preregistered before the data collection using the templet of AsPredicted.org (which can be accessed on https://osf.io/zjfyx). We hypothesised that consumers with promotion orientation are willing to buy and to pay more for foods produced using microbial applications whereas consumers with prevention orientation are not (see the Data Section for the other hypotheses). This study was performed during the COVID-19 pandemic in Europe and, hence, a third objective has been added, being (iii) to assess the effect of the current COVID-19 pandemic on consumers’ attitude towards microbial applications in food production. The latter consideration was added to the study as consumers might associate COVID-19 with potential health risks of using microorganisms in food production, and consumers might also change their diet or purchasing behaviour as a result of the pandemic.

## Materials and methods

### Conceptual framework

Consumer’s food choice problem and WTP analyses are rooted in the random utility theory (RUT) [36]. RUT hypothesises that consumer *i* (*i* = 1, 2, …, *N*) chooses alternative *j* (*j* = 1, 2, …, *J*) if the utility derived from the consumption of alternative *j* (*U*_*ij*_) is greater than the utility derived from the other competing alternative *k* (*U*_*ik*_), for all *j* ≠ *k*. An individual’s latent utility derived from choosing alternative *j* over other alternatives consists of deterministic (observable) and random error (unobservable) components:

$$U_{ij}=\beta X_{ij}+\varepsilon_{ij}$$ (1)

where *β* is a vector of parameters to be estimated, *X*_*ij*_ is a vector of observable factors (i.e. attributes) including personal and socio-demographic characteristics that influences individual *i*’s utility derived from choosing alternative *j*, and *ε*_*ij*_ is the random component capturing the effects of the individual’s unobservable behaviour and variations in preferences, and measurement errors.

In the context of the current study, a consumer switches from consuming a conventional food product produced with chemical applications during the plant production phase to a microbial-based food product that is produced with microbial applications (with lower or no chemical applications) if, and only if, the microbial-based product increases his/her utility of consumption (i.e. if the change in utility is positive), *ceteris paribus* (everything else being equal). Rationally, a consumer will be willing to pay a premium if the change in utility is positive and the increased price does not lower the utility to the base level that is derived from the consumption of the conventional food product [37]. Therefore, the WTP of consumer *i* depends on the change in utility and can be expressed, without loss of generality, as:

$$WTP_{i}=\beta X_{i}+\varepsilon_{i}$$ (2a)

where *WTP*_*i*_ = *WTP*_*ij*_ − *WTP*_*ik*_; *X*_*i*_ = *X*_*ij*_ − *X*_*ik*_ and *ε*_*i*_ = *ε*_*ij*_ − *ε*_*ik*_. In this study, *WTP*_*ij*_ refers to the WTP of consumer *i* for alternative *j* (i.e. the food product that is produced with microbial applications) and *WTP*_*ik*_ refers to the WTP of consumer *i* for alternative *k* (i.e. the conventional food product). The consumers’ latent WTP is measured as an ordinal variable (with five categories; Eq 2b). Respondents were asked to choose their WTP for a food product that is produced with microbial applications compared to a reference non-organic food product from a range of premiums in euro cents. The questions were phrased as “Suppose the current price of fresh potato/wheat bread/tomato sauce is [X] euro/kg. Would you pay a premium for the same amount of food product produced with [X]% less chemical usage due to microbial applications? (i) No, (ii) Yes, I would pay between 1 cent and 10 cents more, (iii) Yes, I would pay between 11 cents and 20 cents more, (iv) Yes, I would pay between 21 cents and 50 cents more, and (v) Yes, I would pay more than 50 cents more”. Therefore, Eq 2a can be rewritten as an ordered logit model:

$$WTP_{i}=\{\begin{array}{l} 0,\;if\;the\;respondent\;is\;not\;WTP\;premium\;\left(WTP=0\;euro\;cent\right)\\ 1,\;if\;the\;respondent\;is\;WTP\;premiums\;of\;1-10\;euro\;cents\\ 2,\;if\;the\;respondent\;is\;WTP\;premiums\;of\;11-20\;euro\;cents\\ 3,\;if\;the\;respondent\;is\;WTP\;premiums\;of\;21-50\;euro\;cents\\ 4,\;if\;the\;respondent\;is\;WTP\;premiums\;of>50\;euro\;cents \end{array}=\beta X_{i}+\varepsilon_{i}$$ (2b)

As WTP likely varies across consumers, personal and socio-demographic characteristics are often included in *X*_*i*_, which normally consists of product characteristics (e.g. with or without microorganisms in the case of the present study). The use of logit models, with a random component, allows to account for individuals’ preference heterogeneity in WTP analyses. However, psychological factors such as consumers’ FCMs, attitude, and environmental and health concerns also influence consumers’ preference and purchasing behaviour heterogeneity [8, 9, 14, 15]. Incorporating psychological factors in the model (Eq 3) allows to explain the underlying consumers’ preference heterogeneity.

**Fig 1:**
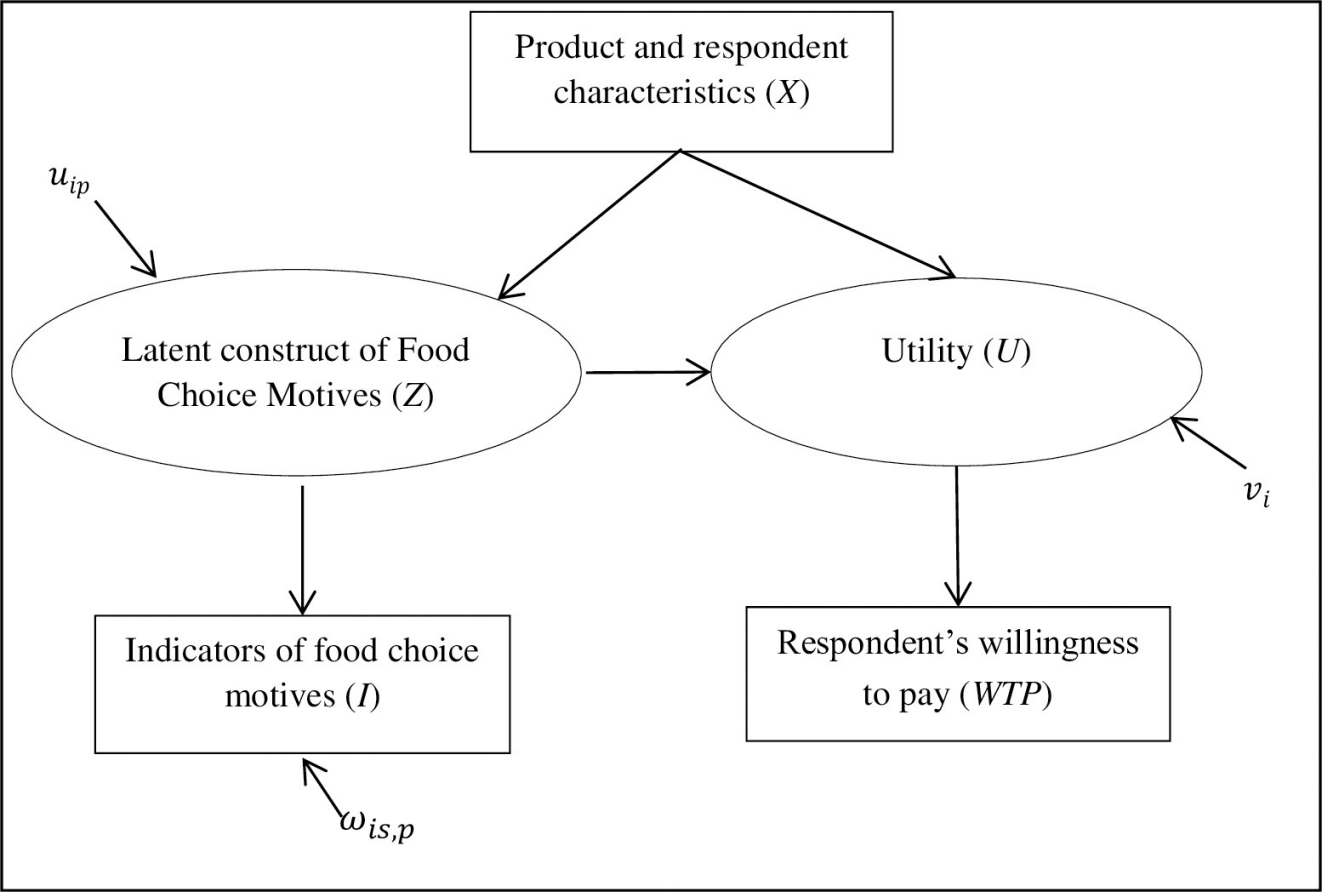
Representation of the willingness to pay and latent variable models used in the analysis. Note: Observed and latent variables are depicted in squares and ellipses, respectively.

The latent variables can be directly included in the WTP model [8, 38], by re-writing Eq 2a as:

$$WTP_{i}=\beta X_{i}+\gamma Z_{i}+v_{i}$$ (3)

where *γ* is a parameter to be estimated, *Z*_*i*_ is a latent variable, and *v*_*i*_ is an independently and identically distributed (IID) error term. Fig 1 visualises the model specifications used in the present study. Including latent variables (e.g. FCM) as explanatory variables in the WTP model (Eq 3) would result in endogeneity bias [39] and measurement error problems [40, 41]. To overcome problems associated with incorporating latent variables, structural equation modelling (SEM) is proposed [40, 42]. The latent variables *Z*_*i*_ are constructed from their indicators as described below.

### Latent variable model

The latent variable model is estimated within the structural equation model using the two-step approach of Anderson and Gerbing [43]. First, a confirmatory factor analysis (CFA) is used to construct the two latent variables, by assessing the relations between the observed indicators and the latent constructs. The promotion- and prevention-oriented FCM latent constructs are derived from the respective observed FCM indicators (Table 1) using the following model:

$$I_{is,p}^{*}=b_{s,p}Z_{is,p}+\omega_{is,p}$$ (4)

where $I_{is,\;p}^{*}$ refers to the s^th^ indicator (s = 1, 2, …, 6 for promotion-oriented and 7, …, 11 for prevention-oriented FCMs as defined in Table 1) for the *p*^th^ latent variable (promotion and prevention oriented FCMs), *b*_*sp*_ is the parameter to be estimated, and *ω*_*is*,*p*_ is the error term and assumed to be normally IID for each indicator and uncorrelated across indicators within a construct. The root mean square error of approximation (RMSEA), the Bentler comparative fit index (CFI) and the standardised root mean square residual (SRMR) measures of goodness-of-fit are used to assess the overall fit of the model, with cut-off values for acceptance of ≤ 0.06, ≥ 0.95 and ≤ 0.08, respectively [44, 45]. The results of the fitted model are used in the second step.

**Table 1:** Food choice motive statements/indicators^a^.

Indicators		Orientation
FCM1	She likes to vary her meals. She is curious about new product options.	Promotion
FCM2	She prefers natural products. She would really like her food fresh from the garden.	Promotion
FCM3	She is grateful for her meal. In her view everything that is edible deserves respect.	Promotion
FCM4	She feels proud of her taste. She believes that her food choices are very attractive.	Promotion
FCM5	She is very mindful of food. She wants to eat sensibly by considering the impacts of her food choice on the environment and on her health.	Promotion
FCM6	She enjoys eating well. In her view every meal should be festive.	Promotion
FCM7	She prefers an ordinary meal. She is happy with existing foods she used to it.	Prevention
FCM8	Food does not bother her. She has no special demands on it such as food safety, healthiness and environmental-friendliness.	Prevention
FCM9	She is a big eater. She loves to have plenty of palatable foods.	Prevention
FCM10	She always sticks to her usual food choice.	Prevention
FCM11	She eats because she has to. Meals are not important to her.	Prevention

^a^ Respondents were asked the question ‘How much your food choice motives resemble those of the person depicted in these 11 statements?’ using a 6-point scale from 1 = ‘Not like me at all’ to 6 = ‘Very much like me’. Source: de Boer *et al*. [9].

In the second stage, the fitted latent constructs are estimated within the SEM as a linear function of product and socio-demographic characteristics:

$$Z_{ip}=\alpha X_{i}+u_{ip}$$ (5)

where *α* is a parameter to be estimated capturing the impact of product, personal and socio-demographic characteristics *X*_*i*_ on the latent variable *Z*_*ip*_ and *p* refers to the latent variable, and *u*_*ip*_ refers to the error term. The error terms are assumed to be normally IID and may correlate across the latent variables (i.e. the error terms of the promotion- and prevention-oriented constructs are allowed to correlate). Product and socio-demographic variables being not statistically significant at critical 20% level are removed one by one from the model, starting with the lowest t-value [46]. Then, Eqs 4 and 5 are estimated simultaneously (using SEM). Finally, Eq 3 is estimated using an order logistic regression model, by using the predicted scores of the latent constructs *Z*_*i*_ as explanatory variables. We have first tried to estimate Eqs 3, 4 and 5 simultaneously within the SEM. However, this attempt has led to a model convergence problem.

### Data

Online surveys were used to collect consumer respondent data for three food products (wheat bread, consumer potatoes and tomato sauce). These food products were selected as part of the EU Horizon 2020 SIMBA project (Sustainable Innovation of Microbiome Applications in Food System) for reflecting the diversity of food value chains in terms of organisation, technology, climatic conditions and consumption patterns across the EU. Three questionnaires corresponding to the three food products were prepared. The questionnaires consisted of four main parts: (1) socio-demographic characteristics (e.g. gender, education, income; Part One), (2) health and environmental concerns related to chemical use in farming, knowledge about microbial applications, perceived microbial health risks and attitude towards microbial applications in food production (Part Two), (3) questions for eliciting a consumer’s WTP for a food product that had been obtained through a microbial-enhanced production system with reduced or no chemical use (Part Three), and (4) questions for eliciting a respondent’s FCMs using de Boer et al.’s [9] FCM questionnaire (Part Four). In addition, the questionnaire had an introduction section containing information sheet about the study and a consent form. The consent form and the information sheet for safeguarding the ethical aspects of this study (e.g. data handling, privacy and potential risks to respondents) were approved by the General Assembly of the SIMBA project as well as the Social Sciences Ethics Committee of Wageningen University prior to distributing the surveys. We collected data primarily from three countries: Germany, Italy and Netherlands. The questionnaires were translated from English into the national languages of these three countries (i.e. German, Italian and Dutch). The questionnaires were distributed online using the Qualtrics software through social media platforms, and via the involved partner institutions in the respective countries (Wageningen University, University of Parma, Natural Resources Institute Finland, and Italian National Agency for New Technologies, Energy and Sustainable Economic Development). In addition to the responses from the three countries, a few responses from Finland and other EU countries were obtained through the support of the SIMBA partner institutions.

Respondents were asked to fill out one of the two questionnaires for their preferred food product: wheat bread- or consumer potato-questionnaire for respondents in Germany and the Netherlands, and wheat bread- or tomato sauce-questionnaire for Italian respondents. Before answering questions regarding microbial applications, respondents were first provided a video explanation [47] of the expected benefits of microbial applications in arable farming during the survey. The Contingent Valuation Method (CVM) was used for eliciting a consumer’s WTP price premium for 20%, 50%, 80% and 100% reduction in chemical use in crop farming as a result of microbial applications as compared to the current conventional intensive farming systems (i.e. each respondent was asked to provide his/her WTP premiums for a 20%, 50%, 80% and 100% reductions in chemical use). Since the actual level of reduction in chemical use due to microbial applications is at experimental (research) stage, these potential levels of reductions were used for covering all possible ranges of reductions. CVM is a commonly applied technique for eliciting consumer WTP for improved food safety and sustainability performances of hypothetical food products [48–50]. Given the April 2020 retail market prices of wheat bread, consumer potatoes and tomato sauce in the respective countries as the reference prices, respondents were asked to choose their WTP from arbitrarily defined sets of price premiums (zero, 1 to 10 cents, 11 to 20 cents, 21 to 50 cents or more than 50 cents per kg of food product). The drawbacks of the CVM and strategies that we followed for mitigating the effects of these drawbacks on our WTP estimates (e.g. using a video explanation for reducing biases associated with the hypothetical product and lack of information) are described in the Discussion section. Table 2 presents the description of variables used in the analysis.

**Table 2:** Description of variables used in the analysis.

Variable name	Description	Measurement
WTP_20%	WTP for a 20% reduction in chemical use in wheat/potato/tomato farming due to microbial application	5 categories: 0 if WTP is zero, 1 if WTP is 1–10 euro cents per kg food product, 2 if WTP is 11–20 euro cents, 3 if WTP is 21–50 euro cents, 4 if WTP is >50 euro cents
WTP_50%	WTP for a 50% reduction in chemical use in wheat/potato/tomato farming due to microbial application	
WTP_80%	WTP for an 80% reduction in chemical use in wheat/potato/tomato farming due to microbial application	
WTP_100%	WTP for a 100% reduction in chemical use in wheat/potato/tomato farming due to microbial application	
Household size	Number of persons in the household	Number
Age ^a^	Age of the respondent in years	Years
Higher education ^b^	Level of education of the respondent	1 = higher education, 0 = otherwise
Gender ^a^	Gender of the respondent	1 = male, 0 = female
Residence	Residence of the respondent	1 = urban, 0 = rural area
Income ^b^	Annual joint household gross income in euro	7-point scale: 1 = <40,000 to 7 = >90,000 euro
Expenditure	Percentage of household gross income spent on food	1 = <5% to 6 = >45%
Consumption frequency	Consuming wheat/potato/tomato products in main meal	1 = once a month or less to 5 = daily
Product type	Type of food products consumed most of the time	1 = organic, 0 = otherwise
Purchasing place	Commonly used purchasing place	1 = supermarket, 2 = farmers shop, 3 = open market, 4 = other
Env’tal concern ^b^	Concerned about the environmental impact of chemical use in agriculture	5-point Likert scale: 1 = strongly disagree to 5 = strongly agree
Health concern ^b^	Concerned about the health risks of chemical residues in food products	5-point Likert scale: 1 = strongly disagree to 5 = strongly agree
Microbial knowledge	Level of knowledge about the use microorganisms in food production	4-point scale: 0 = not knowledgeable to 3 = very knowledgeable
Attitude	Attitude towards the use of microorganisms in food production	5-point Likert scale: 1 = ‘Strongly negative’ to 5 = ‘Strongly positive’
Perceived microbial health risk	Health concern due to the use of microorganisms in food production	4-point scale: 1 = not concerned at all to 4 = very concerned
Potato	Dummy for potato	1 = potato, 0 = otherwise
Tomato	Dummy for tomato	1 = tomato, 0 = otherwise
Germany	Dummy for Germany	1 = Germany, 0 = otherwise
Netherlands	Dummy for Netherlands	1 = Netherlands, 0 = otherwise
Finland	Dummy for Finland	1 = Finland, 0 = otherwise
Other country	Dummy for Other country	1 = Other country, 0 = otherwise
Covid19	Affected by COVID-19 (oneself or someone close to)	1 = yes, 0 = no
Covid19-food	Change diet/food purchasing behaviour due to COVID19	1 = yes, 0 = no
Covid19-microbe	Covid-19 led to attitude change towards using microorganisms in food production	1 = yes, 0 = no

^a^ In the preregistered hypotheses, a negative association with WTP for food stuffs produced with microbial applications was hypothesized.

^b^ A positive association with WTP was hypothesised.

## Results

### Descriptive statistics of respondents’ characteristics

Table 3 summarises the descriptive statistics of the respondent characteristics. Of the total 291 respondents, the majority are female, highly educated, young, urban residents from Italy (148) and Germany (73). The remaining responses are from the Netherlands (35), Finland (12) and other countries (23). One hundred ninety-two responses are for the wheat bread questionnaire whereas 70 and 29 responses are obtained for the tomato sauce and consumer potato questionnaires, respectively. On average, most of the respondents are concerned about the impact of synthetic chemical use in agriculture on their health and the environment (average response of 4 on a 5-point scale) (Table 3). Although the majority of the respondents has little or no knowledge about the potential benefits of microorganisms in food production, most of them have a favourable attitude towards the use of microorganisms in food production (Table 3), with about 77% of them having a positive or very positive attitude towards microbial applications (Table 4). About 46% of the respondents are not concerned at all about any health risk associated with microbial applications in food production. Only 8% of the respondents are (very) concerned about health risks (Table 4). Attitude and perceived health risk are significantly (*p<0*.*01*) negatively correlated (-0.32), implying that consumers who have a favourable attitude towards microbial applications are also those who are not concerned about health risks associated with microbial applications in food production.

**Table 3:** Descriptive statistics of variables^a^.

Variable	Unit	*N*	Mean	Std. Dev.	Min	Max
***Personal and socio-demographic characteristics***						
Household size	Number	286	2.81	1.38	1.00	8.00
Age	Year	275	37.23	13.51	18.00	75.00
Higher education	Yes = 1, No = 0	288	0.81	0.39	0.00	1.00
Gender	Male = 1, Female = 0	278	0.32	0.47	0.00	1.00
Residence	Urban = 1, Rural = 0	291	0.64	0.48	0.00	1.00
Income	Scale 1–7	286	3.17	2.26	1.00	7.00
Expenditure	Scale 1–6	288	2.92	1.04	1.00	6.00
Consumption frequency	Scale 1–5	291	3.97	1.09	1.00	5.00
Product type	Organic = 1, Other = 0	286	0.31	0.46	0.00	1.00
Purchasing place	Category 1–4	291	1.36	0.81	1.00	4.00
Potato	Yes = 1, No = 0	291	0.10	0.30	0.00	1.00
Tomato	Yes = 1, No = 0	291	0.24	0.43	0.00	1.00
Germany	Yes = 1, No = 0	291	0.25	0.43	0.00	1.00
Netherlands	Yes = 1, No = 0	291	0.12	0.33	0.00	1.00
Finland	Yes = 1, No = 0	291	0.04	0.20	0.00	1.00
Other country	Yes = 1, No = 0	291	0.08	0.27	0.00	1.00
Environmental concern	Scale 1–5	262	4.18	0.89	1.00	5.00
Health concern	Scale 1–5	263	3.97	1.01	1.00	5.00
Microbial knowledge	Scale 0–3	285	1.38	1.17	0.00	3.00
Attitude towards microbial use	Scale 1–5	284	4.11	0.85	1.00	5.00
Perceived microbial health risk	Scale 1–4	273	1.64	0.69	1.00	4.00
***COVID-19 related variables***						
Affected by Covid-19	Yes = 1, No = 0	256	0.32	0.47	0.00	1.00
Covid-19 affected food consumption	Yes = 1, No = 0	256	0.38	0.49	0.00	1.00
Covid-19 led to attitude change towards microbial applications	Yes = 1, No = 0	252	0.21	0.41	0.00	1.00

^a^ Refer to Table 2 for the description of variables and measurement.

**Table 4:** Frequency of responses on attitude towards microbial use and perceived health risk associated with microbial applications in food production.

		*Perceived microbial health risk*				
	Scale	Not concerned at all	Somewhat concerned	Concerned	Very concerned	Total
*Attitude*	Strongly negative	0	0	0	1	1
	Negative	0	2	3	0	5
	Neutral	15	36	7	0	58
	Positive	42	56	5	2	105
	Strongly positive	68	32	1	3	104
	Total	125	126	16	6	273

### Summary of WTP survey results

**Fig 2:**
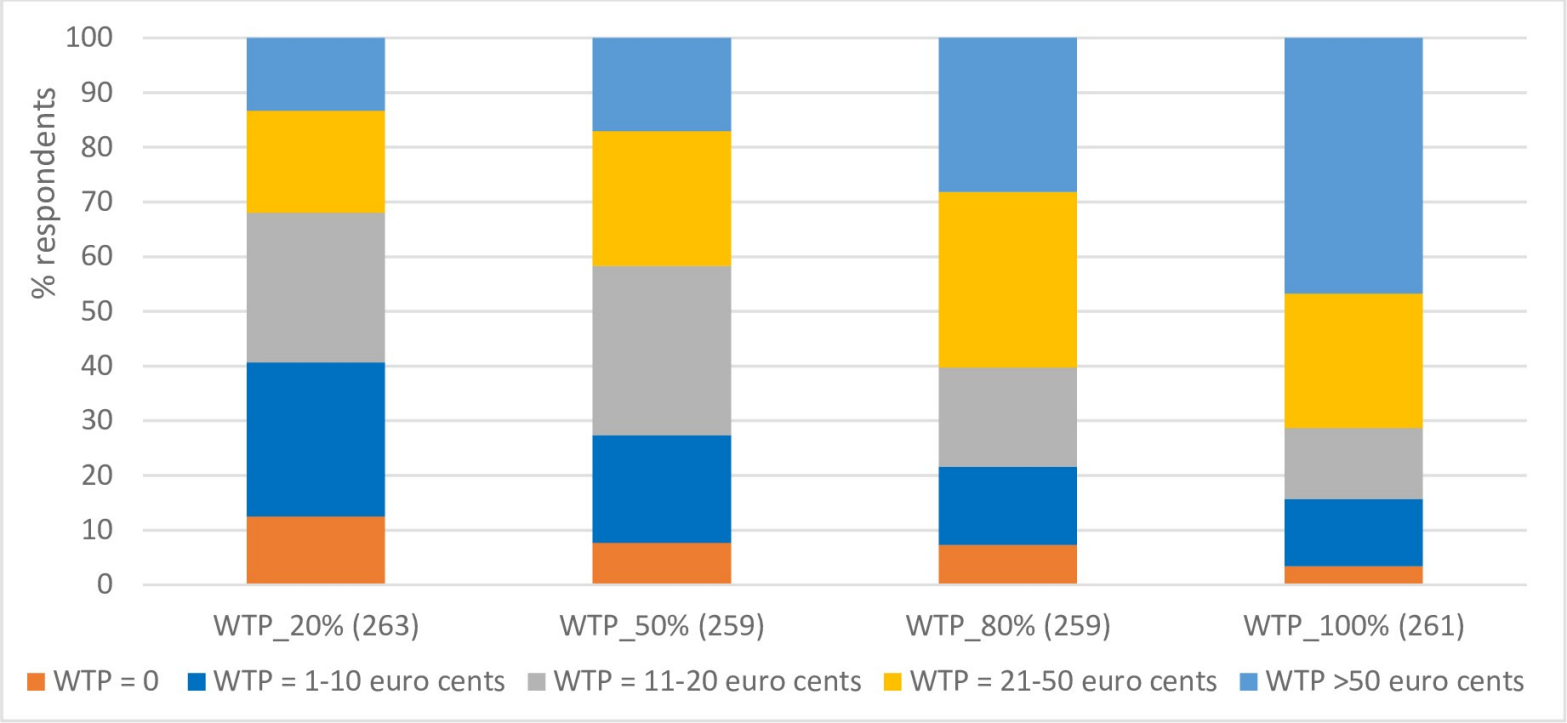
Frequency of responses for WTP for food products that are produced with microbial applications. Note: *WTP_20%*, *WTP_50%*, *WTP_80%* and *WTP_100%* refer to respondents’ WTP premiums for 1 kg of food product (consumer potato/wheat bread/tomato sauce) that is produced with a 20%, 50%, 80% and 100% less synthetical chemical use in primary production by replacement with microbial applications, respectively. The figures in the parenthesis refer to number of respondents.

Fig 2 summarises the responses for consumer’s WTP. For a 20% reduction in synthetical chemical use due to microbial applications (*WTP_20%*), the majority of consumers are willing to pay extra 1 to 10 euro cents (28%) and 11 to 20 euro cents (27%) per kg of wheat bread/tomato sauce/consumer potato. However, about 13% of the respondents are not willing to pay a premium. About 47% of the respondents are willing to pay more than 50 euro cents per kg for a complete replacement of synthetic chemicals with microbial innovations (*WTP_100%*), whereas about 3% of the respondents are not willing to pay a premium in this case. Tables 5 and 6 present the summary statistics of the WTP results per crop type, and per country. Most consumers are willing to pay higher premiums (e.g. >50 euro cents) for wheat bread than for consumer potato and tomato sauce (Table 5). Respondents from Germany are willing to pay more (e.g. >50 euro cents) than respondents from the other study countries (Table 6). These might be due to the fact that wheat bread tends to be more expensive than consumer potato and tomato sauce, and all the German participants responded to the wheat questionnaire. However, these WTP differences across food products and countries are not statistically significant (see the ‘Estimation results of the WTP model’ Section). The distributions (frequency tables) of respondents’ WTP by gender, age and level of education are presented in the S1-S3 Tables of S1 File.

**Table 5:** Frequency of responses for WTP for food products that are produced with reduced use of synthetic chemicals and replacement by microbial applications per crop type.

WTP		Wheat bread		Tomato sauce		Consumer potato	
		Frequency	Percent	Frequency	Percent	Frequency	Percent
***WTP_20%*** ^**a**^	0	23	13.07	7	11.11	3	12.5
	1–10 euro cents	49	27.84	20	31.75	5	20.83
	11–20 euro cents	43	24.43	22	34.92	7	29.17
	21–50 euro cents	34	19.32	8	12.7	7	29.17
	>50 euro cents	27	15.34	6	9.52	2	8.33
	Total	176	100	63	100	24	100
***WTP_50%*** ^**a**^	0	15	8.67	4	6.45	1	4.17
	1–10 euro cents	32	18.5	13	20.97	6	25
	11–20 euro cents	50	28.9	23	37.1	7	29.17
	21–50 euro cents	44	25.43	14	22.58	6	25
	>50 euro cents	32	18.5	8	12.9	4	16.67
	Total	173	100	62	100	24	100
***WTP_80%*** ^**a**^	0	14	8.09	4	6.35	1	4.35
	1–10 euro cents	25	14.45	9	14.29	3	13.04
	11–20 euro cents	31	17.92	11	17.46	5	21.74
	21–50 euro cents	52	30.06	23	36.51	8	34.78
	>50 euro cents	51	29.48	16	25.4	6	26.09
	Total	173	100	63	100	23	100
***WTP_100%*** ^**a**^	0	6	3.43	2	3.23	1	4.17
	1–10 euro cents	18	10.29	9	14.52	5	20.83
	11–20 euro cents	30	17.14	3	4.84	1	4.17
	21–50 euro cents	36	20.57	19	30.65	9	37.50
	>50 euro cents	85	48.57	29	46.77	8	33.33
	Total	175	100	62	100	24	100

^a^ *WTP_20%*, *WTP_50%*, *WTP_80%* and *WTP_100%* refer to respondents’ WTP a premium for 1 kg of food product (i.e. consumer potato/wheat bread/tomato sauce) that is produced with a 20%, 50%, 80% and 100% less synthetical chemical use in primary production by replacement with microbial applications, respectively.

**Table 6:** Frequency of responses for WTP for food products that are produced with microbial innovations by country.

	Finland		Germany		Italy		Netherlands		Others	
***WTP_20%*** ^**a**^	Frequency	Percent	Frequency	Percent	Frequency	Percent	Frequency	Percent	Frequency	Percent
0	2	20	11	16.42	11	8.4	4	11.76	5	23.81
1–10 euro cents	0	0	10	14.93	44	33.59	11	32.35	9	42.86
11–20 euro cents	4	40	10	14.93	40	30.53	14	41.18	4	19.05
21–50 euro cents	2	20	19	28.36	24	18.32	3	8.82	1	4.76
>50 euro cents	2	20	17	25.37	12	9.16	2	5.88	2	9.52
Total	10	100	67	100	131	100	34	100	21	100
***WTP_50%*** ^**a**^										
0	2	20	8	11.94	6	4.69	3	9.09	1	4.76
1–10 euro cents	0	0	10	14.93	26	20.31	5	15.15	10	47.62
11–20 euro cents	3	30	12	17.91	47	36.72	12	36.36	6	28.57
21–50 euro cents	2	20	16	23.88	34	26.56	10	30.3	2	9.52
>50 euro cents	3	30	21	31.34	15	11.72	3	9.09	2	9.52
Total	10	100	67	100	128	100	33	100	21	100
***WTP_80%*** ^**a**^										
0	3	30	7	10.45	5	3.88	2	6.25	2	9.52
1–10 euro cents	0	0	9	13.43	19	14.73	5	15.63	4	19.05
11–20 euro cents	3	30	8	11.94	22	17.05	6	18.75	8	38.1
21–50 euro cents	1	10	17	25.37	49	37.98	11	34.38	5	23.81
>50 euro cents	3	30	26	38.81	34	26.36	8	25	2	9.52
Total	10	100	67	100	129	100	32	100	21	100
***WTP_100%*** ^**a**^										
0	0	0	4	5.97	3	2.33	2	5.88	0	0
1–10 euro cents	2	20	5	7.46	16	12.4	5	14.71	4	19.05
11–20 euro cents	1	10	9	13.43	13	10.08	2	5.88	9	42.86
21–50 euro cents	3	30	12	17.91	34	26.36	13	38.24	2	9.52
>50 euro cents	4	40	37	55.22	63	48.84	12	35.29	6	28.57
Total	10	100	67	100	129	100	34	100	21	100

^a^ *WTP_20%*, *WTP_50%*, *WTP_80%* and *WTP_100%* refer to respondents’ willingness to pay a premium for 1 kg of food product (i.e. consumer potato/wheat bread/tomato sauce) that is produced with a 20%, 50%, 80% and 100% less synthetical chemical use in primary production by replacement with microbial applications, respectively.

### Estimation results of the latent variable model

**Fig 3:**
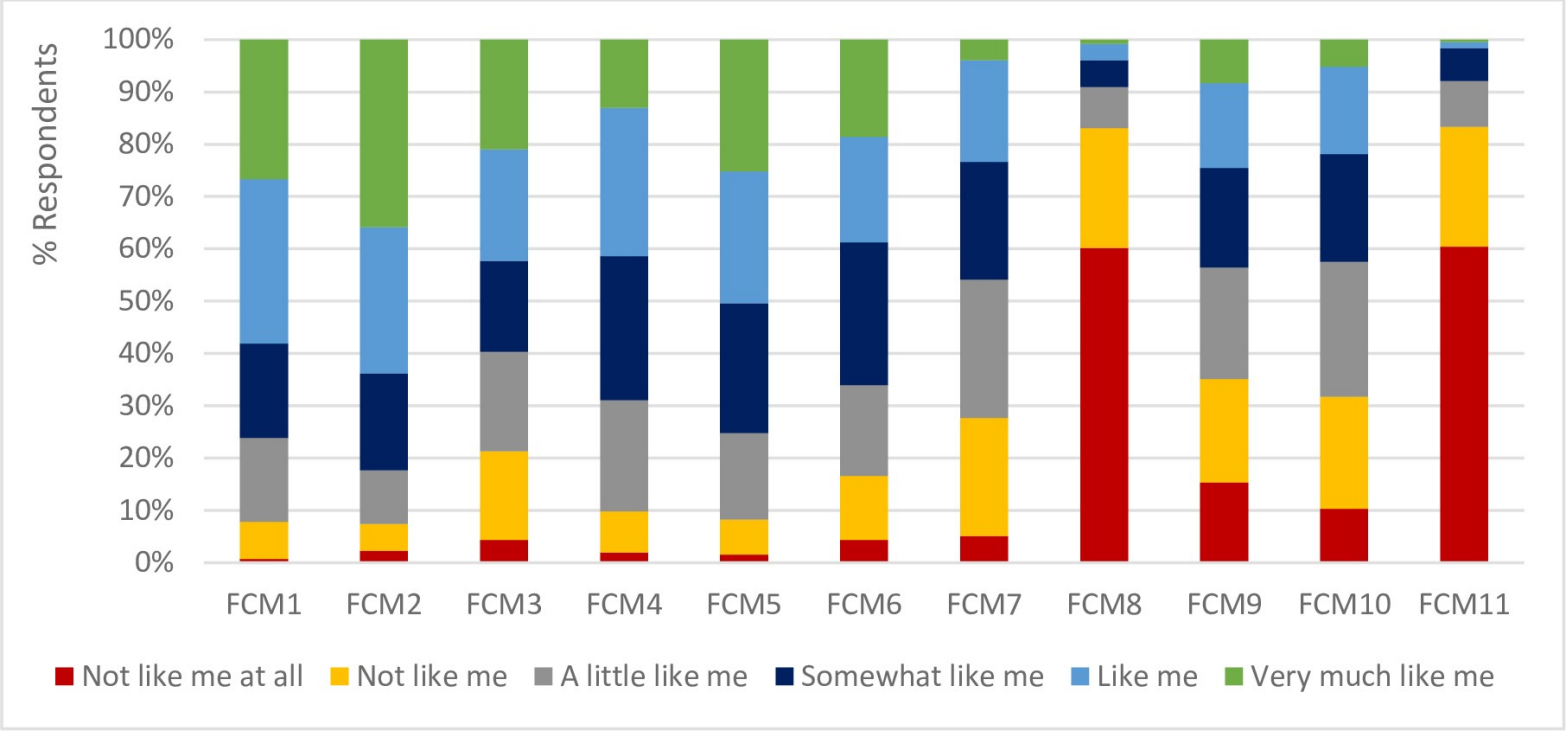
Frequency of responses for the food choice motive indicators. Refer to Table 1 for the complete statements for FCM1 to FCM11.

The results of the FCM survey (Fig 3) show that most of respondents replied ‘*Very much like me*’ or ‘*Like me*’ to the promotion-oriented statements (Table 1) whereas most of them replied ‘*Not like me at all*’ or ‘*Not like me*’ to the prevention-oriented statements.

Table 7 presents the estimation results of the latent variable model (Eqs 4 and 5). The promotion- and prevention-oriented FCM latent constructs are derived from the respective observed FCM indicators (Table 1) using CFA. Indicators that are statistically insignificant (and with the smallest coefficient in Eq 4) (S4 Table in S1 File) are iteratively excluded from the construction of the two latent variables until at least two of the three model goodness-of-fit measures are satisfied. This is reached when indicator *FCM3* is excluded from the construction of the promotion-oriented construct, and indicators *FCM7*, *FCM9* and *FCM10* from the prevention-oriented construct (Table 7). The excluded indicators do not explain variations in the respective latent constructs since most of the respondents replied ‘*A little like me*’ or ‘*Somewhat like me*’ to these indicators (Fig 3). The results of the measurement model from the simultaneous estimation of Eqs 4 and 5 show that the included indicators have a significant association with the respective latent constructs (Table 7). The RMSEA and SRMR measures of model goodness-of-fit indicate that the indicators used in the latent variables’ construction are acceptable in defining the constructs. The significant covariance between the error terms of the two constructs indicate that the unexplained variance is shared between the two latent variables.

**Table 7:** Estimation results from the latent variable model^a^.

	Promotion oriented		Prevention oriented	
***Structural model***	Coefficient	Std. Err.	Coefficient	Std. Err.
Household size	0.13	0.08	-0.15*	0.09
Age	0.15**	0.07		
Higher education			-0.15**	0.07
Gender	-0.09	0.07	0.12	0.08
Residence	0.11	0.08	-0.18**	0.08
Income	-0.09	0.07		
Expenditure			0.20***	0.07
Consumption frequency			0.13*	0.08
Product type	0.15**	0.08	0.15*	0.09
Purchasing place	0.11*	0.07		
Potato			0.15**	0.08
Germany	-0.18**	0.09	0.15	0.09
Netherlands	-0.11	0.08	0.21**	0.09
Other country			0.13	0.08
Environmental concern			-0.25***	0.07
Health concern	0.33***	0.07		
Attitude towards microbial application	0.19***	0.07	-0.16*	0.07
***Measurement model***				
FCM1	0.42***	0.07		
FCM2	0.72***	0.05		
FCM4	0.50***	0.07		
FCM5	0.86***	0.03		
FCM6	0.43***	0.07		
FCM8			0.86***	0.05
FCM11			0.68***	0.06
***Goodness-of-fit measures***				
RMSEA	**0.057** ^b^			
CFI	0.83 ^b^			
SRMR	**0.044** ^b^			
***Error term covariances***				
Promotion oriented	0.60	0.06		
Prevention oriented	-0.68***	0.08	0.73	0.07

^a^ *N* = 160.

^b^ The cut-off values for acceptance of the goodness-of-fit of the specified model are < = 0.06 for RMSEA, > = 0.95 for CFI and < = 0.08 for SRMR. The RMSEA and SRMR measures of model goodness-of-fit indicate that the indicators used in the latent variables’ construction are acceptable in defining the constructs.

Likelihood ratio test of model vs. saturated: Chi^2^(143) = 218.49***.

***, **, *Significant at 1%, 5% and 10% critical levels, respectively.

The results of the structural model show that older people, organic food consumers, those who purchase elsewhere than from supermarkets, and consumers who are concerned about the impact of synthetic chemical use in agriculture on the environment and their health are more promotion-oriented (Table 7). The negative coefficient of the dummy variable “Germany” implies that respondents from Germany are 18% less promotion-oriented than those from Italy (Table 7). Consumers who spend relatively more of their income on food, frequent consumers of the product under consideration, organic food consumers, potato derived food consumers (compared to wheat derived food consumers), and respondents from the Netherlands and Other country (compared to Italian consumers) are more prevention-oriented consumers. On the other hand, consumers with a large household size, who are highly educated, from urban areas and those who are concerned about the environmental impact of chemical use in agriculture are less prevention-oriented. For example, consumers with higher education are 15% less prevention-oriented than consumers without higher education. The complete estimation results of the latent variable model, where all the eleven indicators are included in the measurement model, can be seen in S4 Table in S1 File. The results are quantitatively and qualitatively similar to the results presented in the main text.

The descriptive statistics of the predicted factor scores of the promotion- and prevention-oriented FCM latent constructs are shown in Table 8. The average predicted factor score is positive for the promotion-orientation whereas it is negative for the prevention-orientation FCM latent construct. A one unit increase in the value of the promotion-oriented construct implies a change from less to more promotion-orientation. However, since the average predicted factor score for the prevention-oriented construct is negative, an increase in the predicted factor score implies a change from being more prevention- to less prevention-orientation (i.e. a reverse scale is applied in this empirical application). The correlation between the predicted factor scores of the promotion- and prevention-oriented FCM latent constructs (-0.69) is statistically significant at the 1% level.

**Table 8:** Descriptive statistics of predicted factor scores of the latent variables.

Latent variables	Mean	Std. Dev.	Minimum	Maximum
Factor score of promotion-oriented construct	1.64	0.50	0.10	2.54
Factor score of prevention-oriented construct	-1.32	0.84	-2.23	2.28

### Estimation results of the WTP model

Before estimating the WTP model (Eq 3), we check the correlations between the FCM latent constructs and the WTP outcomes (Table 9). The results indicate that there exist, respectively, a positive and negative significant correlation between consumers’ WTP, and promotion- and prevention-oriented FCMs. This implies that highly promotion-oriented and less prevention-oriented consumers are willing to pay premiums for food products that are produced with microbial applications to replace synthetic chemicals. These consumers’ WTP increases with the reduction in chemical use from 20% to 100% (Table 9). The significant positive and negative coefficients of the variables on health and environmental concerns, respectively for promotion- and prevention-oriented constructs (Table 7), imply that health concerned promotion-oriented consumers and environment concerned prevention-oriented consumers are likely to pay more for food products that are produced in microbe-enhanced production systems. Similarly, the significant positive and negative coefficients of the variable on attitude towards microbial application respectively for promotion- and prevention-oriented consumers (Table 7) imply that consumers who have a favourable attitude towards microbial applications in food production are also likely to pay a premium for these food products.

**Table 9:** Correlation between consumers’ WTP and their FCM orientation.

WTP ^a^	Promotion oriented construct	Prevention oriented construct
*WTP_20%*	0.16**	-0.05
*WTP_50%*	0.18***	-0.09
*WTP_80%*	0.25***	-0.10*
*WTP_100%*	0.32***	-0.23***

^a^ *WTP_20%*, *WTP_50%*, *WTP_80%* and *WTP_100%* refer to respondents’ willingness to pay a premium for 1 kg of food product (i.e. consumer potato/wheat bread/tomato sauce) that is produced with a 20%, 50%, 80% and 100% less chemical use in farming due to microbial applications, respectively.

***, **, *Significant at 1%, 5% and 10% critical levels, respectively.

Contrary to our preregistered hypotheses, the maximum likelihood estimation results of the ordered logistic WTP model show that the explanatory variables are not statistically significant and do not have the expected signs (S5 and S6 Tables in S1 File). This might be due to the small sample size (and the subsequent lack of variation in the dataset), as there are only 213 observations over the four WTP categories (S5 Table in S1 File). As a result, we re-estimated the WTP model (Eq 3) as a binary logistic model, where *WTP* = 1 if a respondent is willing to pay at least 1 euro cent premium per kg of food product and 0 otherwise. The estimation results and the marginal effects are presented in Table 10. Marginal effect indicates the effect of a change in an explanatory variable on the predicted probability that a consumer is willing to pay, everything else being equal. The included explanatory variables are jointly significant in explaining the variation in the predicted probability of WTP (i.e. the null hypothesis of jointly zero slope coefficients is rejected by the Wald test). The average predicted probabilities of WTP for the food products that are produced with microbial applications are 0.88 and 0.91 for *WTP_20%* and *WTP_50%*, respectively. This suggests a strong probability that an average consumer is willing to pay a price premium. The “Margins” command in STATA has been used to compute the predicted probability of WTP, keeping all the explanatory variables of the model at their means.

**Table 10:** Maximum likelihood estimation results of the logistic WTP model and marginal effects. (*WTP* = 1 if a respondent is willing to pay at least 1 euro cent per kg of a food product, 0 otherwise)^a^.

	Logistic regression (probabilities)				Marginal effects			
	*WTP_20%*		*WTP_50%*		*WTP_20%*		*WTP_50%*	
Variables	Coef.	SE	Coef.	SE	Coef.	SE	Coef.	SE
Promotion oriented FCM	3.31***	1.00	4.01***	1.42	0.26***	0.08	0.27***	0.09
Prevention oriented FCM ^b^	1.91***	0.69	3.25***	1.11	0.15***	0.05	0.22***	0.07
Attitude	0.17	0.41	0.62	0.56	0.01	0.03	0.04	0.04
Microbial knowledge	0.14	0.26	-0.39	0.47	0.01	0.02	-0.03	0.03
Perceived microbial health risk	-0.25	0.41	0.21	0.62	-0.02	0.03	0.01	0.04
Environmental concern	0.42	0.32	0.61*	0.34	0.03	0.02	0.04*	0.02
Health concern	0.13	0.30	-0.15	0.39	0.01	0.02	-0.01	0.03
Household size	-0.30*	0.19	0.02	0.29	-0.02*	0.02	0.00	0.02
Age	0.01	0.03	-0.03	0.04	0.00	0.00	0.00	0.00
Higher education	0.84	0.91	0.66	1.53	0.07	0.07	0.04	0.10
Gender	-0.30	0.57	-0.14	0.88	-0.02	0.04	-0.01	0.06
Residence	-2.14**	0.89	NA	NA	-0.17***	0.06	NA	NA
Income	0.24*	0.13	0.02	0.19	0.02*	0.01	0.00	0.01
Expenditure	0.49*	0.26	0.25	0.34	0.04*	0.02	0.02	0.02
Consumption frequency	0.77***	0.31	0.47	0.35	0.06***	0.02	0.03	0.02
Product type	-0.98	0.63	-1.97**	0.95	-0.08	0.05	-0.13**	0.07
Purchasing place	-0.45	0.36	-0.42	0.49	-0.03	0.03	-0.03	0.03
Potato	1.39	1.18	0.64	1.25	0.11	0.09	0.04	0.08
Tomato	-0.10	0.92	0.01	1.39	-0.01	0.07	0.00	0.09
Germany	0.05	0.91	0.51	1.52	0.00	0.07	0.03	0.10
Netherlands	-1.32	1.02	-2.13*	1.23	-0.10	0.08	-0.14*	0.07
Finland	*NA*	*NA*	*NA*	*NA*	*NA*	*NA*	*NA*	*NA*
Other country	-1.81**	0.89	0.02	1.23	-0.14**	0.07	0.001**	0.08
Constant	-5.15	3.29	-3.87	4.72				
*Goodness-of-fit*								
Observations	213		140					
Log likelihood	-54.63		-32.32					
Wald Chi^2^	35.60**		43.24***					
Pseudo R^2^	0.29		0.25					

^a^ Estimated with robust standard errors.

^b^ Here a reverse scale is applied. An increase in the predicted factor score implies a change from being more prevention-oriented to less prevention-oriented consumer, since the average predicted factor score is negative (see Table 8).

Note: *NA* refers to dropped variable from the regression due to perfect collinearity with the dependent variable.

***, **, *Significant at 1%, 5% and 10% critical levels, respectively.

As can be seen from the coefficients of the marginal effects (Table 10), the promotion- and prevention-oriented FCM constructs have positive associations with the predicted probabilities of WTP, which confirm our hypotheses. On average, a one unit increase in the predicted factor score of the promotion-oriented construct is associated with a 26% increase in the predicted probability of WTP for food products that are produced with 20% less chemical use, *ceteris paribus* (i.e. highly promotion-oriented consumers are more likely to pay premiums) (Table 10). On the other hand, a one unit increase in the predicted factor score of the prevention-oriented construct is associated with a 15% increase in the predicted probability of WTP for food products that are produced with 20% less chemical use, *ceteris paribus* (Table 10). This implies that less prevention-oriented consumers (who responded ‘*Not like me at all*’ or ‘*Not like me*’ for the prevention FCM statements) are more likely to pay premiums for food products that are produced with microbial applications compared to highly prevention-oriented consumers (who responded ‘*Very much like me*’ or ‘*Like me*’ for the prevention FCM statements). Since the average precited factor score for the prevention-oriented construct is negative, an increase in the predicted factor score implies a change from being more prevention- to less prevention-orientation. *Ceteris paribus*, relatively environmentally concerned consumers are also likely to be willing to pay more premiums (positive marginal effects) for a 50% reduction in chemical use compared to less concerned consumers. Consumers with higher income and those who spend higher percentage of their income on food are more likely to pay premiums. Frequent consumers (e.g. those who consume daily) of a given food product are more likely to be willing to pay premiums for food products that are produced with microbial applications compared to those who consume occasionally. *Ceteris paribus*, consumers with a bigger household size, who live in urban areas, consume organic food products (compared to conventionally produced foods), and those from the Netherlands (compared to from Italy) are less likely to be willing to pay premiums. Against our hypotheses, health concern, gender, age and education do not have statistically significant associations with WTP.

### Effect of COVID-19 pandemic on willingness to buy microbial-enhanced food

The descriptive statistics of the COVID-19 related variables are summarised in Table 3. About 32% of the 256 respondents indicated that they (or someone close to them) have been affected by the pandemic. About 38% of the respondents have also indicated that they changed their diet or their food purchasing behaviour as a result of the pandemic. Examples of the changes that the respondents indicated ‘to stay healthy’ include consuming more plant-based (e.g. vegetables, fruits) instead of animal-based food sources, organic and local products, and reducing frequency of purchasing food, and more of cooking at home (instead of eating outside/restaurants). The survey results showed that the pandemic has also influenced consumer’s attitudes towards the use of microbial applications in food production. About 21% of the respondents stated that they would change their positive attitude towards the use of microorganisms in food production due to the COVID-19 virus. Table 11 presents the correlations between COVID-19 related variables and consumers’ willingness to buy (WTB) food products that are produced with microbial applications. There is no statistically significant correlation between WTB and Covid-19 related variables. However, the small positive associations amongst the COVID-19 related variables are statistically significant.

**Table 11:** Correlations between Covid-19 related variables and consumers’ willingness to buy food products that are produced with microbial applications.

	Unit	WTB	Covid19	Covid_food
Willing to buy microbial-based food products (WTB)	Yes/No	1		
Affected by COVID-19 (oneself/someone close to) (Covid19)	Yes/No	-0.01	1	
Change diet or food purchasing behaviour due to COVID-19 (Covid_food)	Yes/No	0.06	0.13**	1
COVID-19 enhanced attitude changes towards microbial use in food production	Yes/No	0.09	0.15**	0.14**

**Significant at 5% critical level.

## Discussion

To date, no other studies on consumers’ WTP for food products that are produced with microbial applications are available and, therefore, below we compared the results of the present study with results from the literature for pesticide-free, organic and related food products. A meta-analysis by Florax *et al*. [51] reported that the WTP for reduced pesticide risk exposure is 80% and 15% greater for high and medium risk levels compared with low risk levels, respectively. They also reported that income does not have a significant effect on WTP for reduced pesticide risk exposure. In line with these results, in the present study, consumers’ WTP increases with the increase in the reductions of chemical use, and income has no statistically significant effect on WTP for food products that are produced with microbial applications. Bernard and Bernard [52] found that United States’ consumers are willing to pay premiums of up to $40 cents per kg of organic potatoes compared with conventionally produced potatoes. In the same study, they reported that consumers are willing to pay up to $28 cents per kg of potatoes that are produced without pesticide applications compared to conventional potatoes. Bernard and Bernard [52] concluded that consumers’ WTP for the ‘no pesticide’ component of organic production ($28 cents) is significantly higher than their WTP for the ‘non-genetically modified’ component ($14 cents). In the present study, about 71% of the respondents are willing to pay premiums of at least 21 euro cents per kg of consumer potatoes that are produced without chemical applications (of which 33% are willing to pay at least 50 euro cents more).

The present study was carried out on hypothetical food products and shows that some drivers for consumer WTP in the case of organic produced products are significant also for products produced with microbial inputs, while other drivers are not. This aspect needs further investigation in future studies, when more evidence is available about the effectiveness of actual microbial products in reducing/substituting conventional inputs by farmers. A recent review by Katt and Meixner [53] categorised the drivers of consumers’ WTP for organic products into: consumer-related (e.g. age, income, health and environmental concerns), product-related (e.g. price, food safety, locality, involvement), and purchasing venue-related (e.g. type of store, convenience/ proximity to consumer) factors. In the present study, we have included several factors from each of these three categories in explaining the variations in WTP amongst consumers. In line with the results of our study, Katt and Meixner [53] reported that most studies found positive associations between environmental concern and WTP for organic food products. According to the authors, this might be explained by the consumers perceptions that organic food is more environmentally friendly and chemical use in conventional farming is harmful to the environment. Although health concerns are not statistically significant in influencing WTP in our study, most studies about organic products found significant associations as reported by Katt and Meixner [53]. However, our survey results showed that most of the respondents are concerned about the impact of synthetic chemical use in agriculture on their health and the environment (Table 3), which is in line with results from EU-wide surveys [19]. Young, female, highly educated and high-income earning consumers are reported to be willing to pay more for sustainable and healthy food products [54, 55]. Similarly, the results of the present study (Table 10) show that age, gender, education and income have the expected associations with WTP for foods produced with 20% less chemical use. However, with the exception of income, the associations of these socio-demographic factors with WTP are not statistically significant. This might be caused by the lack of variation, since our sample consists of mainly female (68%), young (average of 37 years old) and highly educated (81% with higher education) consumers. Given our sample, the absolute WTP might be overestimated. However, our sample is assumed to be representative of the population of interest for plant-based food sources (i.e. female, young, and highly educated consumers who are reported in the literature to be willing to pay more for sustainable and healthy food products). Therefore, the higher WTP results of the present study (see the *Summary of WTP Survey Results* Section) might be due to the composition of our sample.

Meixner and Katt [56] reported that “individuals who are more affected by the COVID-19 pandemic are becoming more price sensitive” in the United States for beef attributes (e.g. origin, food safety). The results of the present study showed that about 38% of the respondents changed their diet or their food purchasing behaviour as a result of the COVID-19 pandemic. The survey results also showed that the pandemic has influenced some consumers’ attitudes towards the use of microbial applications in food production (21%) although the association with consumers’ willingness to buy food products that are produced in microbial-enhanced production systems is not statistically significant.

Incorporating psychological (attitudinal) factors in consumers’ food choice analyses increases our understanding of consumers’ behaviour of purchasing and food choice decisions [8, 15, 41]. Specifically, consumers’ attitudinal orientations (i.e. FCMs) are identified as the key drivers of consumers’ choice decisions for new food products [8, 9, 13], like microbial-enhanced products as in the present study. Our FCM survey results showed that most of respondents replied ‘*Very much like me*’ or ‘*Like me*’ to the promotion-oriented statements, and ‘*Not like me at all*’ or ‘*Not like me*’ to the prevention-oriented statements. This implies that the majority of the respondents are promotion-oriented consumers in relation to their food involvement, who are open to taste new food products [8, 9]. Alemu *et al*. [8] incorporated FCMs as one latent variable for explaining Kenyan consumers’ preferences for insect-based food products, and concluded that the latent FCM construct is one of the significant drivers of consumers’ preferences for these products. Since consumers’ food choices are influenced by two distinct attitudinal orientations [9], in the present study, we incorporated FCMs as two latent variables (promotion- and prevention-oriented constructs) in the WTP model. In line with the results of Alemu *et al*. [8], the promotion-oriented FCM construct has a positive significant effect on WTP for food products that are produced with microbial applications. These results imply that food products that are produced with microbial-enhanced production systems could be commercialised (since the majority of the respondents are promotion-oriented and are concerned about the environmental impacts of chemical inputs).

For eliciting the consumer WTP, this study applied CVM over other competing methods (e.g. discrete choice or field experiments or double-bounded dichotomous choice within the CVM) for the following reasons. First, CVM allows to elicit consumer WTP for hypothetical food products in a non-market situation. Since food products that are produced in microbial-enhanced production systems do not exist on the market, we could not apply other methods that involve an actual purchasing situation. Second, it is difficult to define and communicate ‘concrete’ attributes for food products that are produced in microbial-enhanced production systems, for example, to apply choice experiments. Since the potential benefits of microbial applications in food production are at a research level (e.g. improving soil quality and health, reducing chemical use, improving human health through improved product quality), it is difficult to translate these potential benefits into concrete product attributes to design choice sets. Third, the use of double-bounded dichotomous choice CVM in the case of the present study where we elicited a consumer’s WTP premiums for a 20%, 50%, 80% and 100% reductions in chemical use would increase the response burden exponentially (i.e. a respondent would require to respond to four standard WTP questionnaires in the context of a double-bounded dichotomous CVM). Although the CVM allows to elicit consumer WTP directly without purchasing the product, the method has shortcomings that potentially raise concerns about the reliability of estimates. First, respondents may overstate their true WTP since they do not face actual budget constraints [57]. The CVM, however, performs well in cases where the hypothetical situation is similar to a familiar market choice situation [46]. Second, consumers may have little knowledge about “the risks involved and therefore they may give a wrong monetary evaluation of the benefit from risk avoidance” [50]. Informing consumers about the risks involved during the elicitation is recommended to reduce biases associated with lack of information [50]. In the present study, these limitations were taken into account during the data collection. Rather than asking consumers how much premium they are willing to pay, they were asked to choose from a realistic range of premiums in euro cents per kg food product (0, 1–10, 11–20, 21–50, >50). Furthermore, based on Eurobarometer [19], European consumers are known to be well-informed about the impacts of using chemical inputs in farming on the environment and their health, which reduces biases associated with lack of information. Finally, consumers were provided a video explanation of the expected benefits of microbial applications in farming during the survey, which may reduce biases associated with the hypothetical product and lack of information.

Although the magnitude of our WTP estimates is plausible and consistent with existing studies on WTP of alternative primary production methods, we remain cautious about the policy implications because of the limitations of the CVM that we applied, specifically, the response bias of the WTP estimates. A meta-analysis by Florax et al. [51] showed that WTP estimates from CVM are higher than estimates from other stated preference approaches based on revealed preferences and choice experiments. Moreover, the video explanations that we used during the survey for introducing the potential benefits of microbial applications in food production may have influenced respondents’ perceptions, potentially leading to a response bias of the WTP estimates. This video was however deemed necessary since the percent of the consumers having knowledge about microbial applications in arable farming was expected to be very low. Further studies could conduct rigorous analyses using ‘real’ products that are produced with microbial applications, and by applying other approaches such as double-bounded dichotomous choice, revealed preference and choice experiments.

## Conclusions and recommendations

A transition towards a more plant-based diet is a crucial step for transitioning towards a sustainable and healthy food system. The consumers’ interest in plant-based diet, particularly within young and female consumers, is growing due to the perceived environmental and public health benefits of plant-based food sources (e.g. lentils and beans) compared to animal-based food sources (e.g. red meat) [6, 58, 59]. This study assessed the consumers’ WTP for plant-based food products that are produced with microbial applications. Using CVM, the empirical application focused on 291 consumers, primarily from Italy, Germany and the Netherlands. We also evaluated the behavioural and socio-economic characteristics that are associated with the WTP. Results showed that most consumers, about 77% of the respondents, have a positive attitude towards microbial applications in food production. Potential consumers of food products that are produced in microbial-enhanced production systems would be willing to pay premiums of at least 11 euro cents per kg of food products. The amount of consumer WTP increases with the level of reductions of chemical use. The majority of the respondents are shown to have a promotion orientation in relation to their food involvement, and are found to be more likely to be willing to pay price premiums. Relatively, environmentally concerned consumers are also found to be more likely to be willing to pay premiums, whereas health concerned consumers are not. The results of this study imply that promotion-oriented and environmentally concerned consumers could be potential buyers of food products that are produced in microbial-enhanced production systems. The results also suggested that about 21% of the respondents would change their positive attitude towards the use of microorganisms in food production due to the COVID-19 pandemic. This study contributes to a better understanding of consumers’ attitude and perceived risks towards food products obtained using microbial applications. The results provide insights for identifying potential buyers of plant-based food products that are produced using microbial applications and to set prices according to the levels of consumers’ WTP.

We have several recommendations for future research. First, further studies should conduct rigorous analyses using ‘real’ products that are produced with microbial applications, and by applying complementary approaches (e.g. revealed preferences and field experiments). This is an essential step when the benefits and risks of microbial applications will be accurately quantified. Second, asking consumers to make purchases using virtual supermarket platforms [60] where respondents are incentivised to take virtual trips and spend tokens/real money on actual food baskets of conventional food products and food products obtained using microbial applications would be an effective approach for eliciting a consumer’s WTP. Third, we suggest to follow up on this study after the COVID-19 pandemic, as risk attitudes and subsequent WTP may shift hereafter. Finally, we recommend to investigate the production cost and willingness of farmers of microbial applications. Such a production-oriented approach complements the consumer-oriented approach of the current paper.

## Supporting information

S1 File (DOCX)

## References

[1] Tobi RC, Harris F, Rana R, Brown KA, Quaife M, Green R. Sustainable diet dimensions. Comparing consumer preference for nutrition, environmental and social responsibility food labelling: a systematic review. Sustainability. 2019 Jan;11(23):6575. 10.3390/su11236575 PMC7616258 39035350

[2] Evans D, Southerton D, McMeekin A. Sustainable consumption, behaviour change policies and theories of practice. 2012. In: Warde A and Southerton D (eds) The Habits of Consumption. Helsinki: Helsinki Collegium for Advanced Studies, pp. 113–129.

[3] IPCC, Intergovernmental Panel on Climate Change. 2019. Climate Change and Land: An IPCC special report on climate change, desertification, land degradation, sustainable land management, food security, and greenhouse gas fluxes in terrestrial ecosystems. 2019. Accessed on 30 June 2021 from https://www.ipcc.ch/site/assets/uploads/2019/11/SRCCL-Full-Report-Compiled-191128.pdf.

[4] FAO, Food and Agriculture Organization. AQUASTAT database: FAO’s Global Information System on Water and Agriculture. 2021. Accessed on 30 June 2021 from http://www.fao.org/aquastat/statistics/query/results.html.

[5] FAO, Food and Agriculture Organization. Feeding people, protecting the planet: FAO and the GEF: partners in action. 2018. Accessed on 30 June 2021 from https://www.thegef.org/sites/default/files/publications/FAOandGEFpub_062018.pdf.

[6] Springmann M, Clark M, Mason-D’Croz D, Wiebe K, Bodirsky BL, et al. Options for keeping the food system within environmental limits. Nature. 2018 Oct;562(7728):519–25. doi: 10.1038/s41586-018-0594-0 30305731

[7] Siegrist M, Hartmann C. Consumer acceptance of novel food technologies. Nature Food. 2020 Jun;1(6):343–50. 10.1038/s43016-020-0094-x 37128090

[8] Alemu MH, Olsen SB. Linking consumers’ food choice motives to their preferences for insect-based food products: An application of integrated choice and latent variable model in an African context. Journal of Agricultural Economics. 2019 Feb;70(1):241–58.

[9] De Boer J, Hoogland CT, Boersema JJ. Towards more sustainable food choices: Value priorities and motivational orientations. Food Quality and Preference. 2007 Oct 1;18(7):985–96.

[10] Prescott J, Young O, O’neill L, Yau NJ, Stevens R. Motives for food choice: a comparison of consumers from Japan, Taiwan, Malaysia and New Zealand. Food quality and preference. 2002 Oct 1;13(7–8):489–95.

[11] Eertmans A, Victoir A, Vansant G, Van den Bergh O. Food-related personality traits, food choice motives and food intake: Mediator and moderator relationships. Food Quality and Preference. 2005 Dec 1;16(8):714–26.

[12] Steptoe A, Pollard TM, Wardle J. Development of a measure of the motives underlying the selection of food: the food choice questionnaire. Appetite. 1995 Dec 1;25(3):267–84. doi: 10.1006/appe.1995.0061 8746966

[13] de Boer J, Schösler H, Boersema JJ. Motivational differences in food orientation and the choice of snacks made from lentils, locusts, seaweed or “hybrid” meat. Food Quality and Preference. 2013 Apr 1;28(1):32–5.

[14] Wu X, Hu B, Xiong J. Understanding heterogeneous consumer preferences in Chinese milk markets: A latent class approach. Journal of Agricultural Economics. 2020 Feb;71(1):184–98.

[15] Ashok K, Dillon WR, Yuan S. Extending discrete choice models to incorporate attitudinal and other latent variables. Journal of marketing research. 2002 Feb;39(1):31–46.

[16] EC, European Commission. From Farm to Fork: Our food, our health, our planet, our future. 2020. Retrieved on 06 July 2020 from https://ec.europa.eu/commission/presscorner/detail/en/fs_20_908

[17] Boobis AR, Ossendorp BC, Banasiak U, Hamey PY, Sebestyen I, Moretto A. Cumulative risk assessment of pesticide residues in food. Toxicology letters. 2008 Aug 15;180(2):137–50. doi: 10.1016/j.toxlet.2008.06.004 18585444

[18] Hamilton D, Crossley S (Eds.). Pesticide residues in food and drinking water: human exposure and risks. 2004. John Wiley & Sons.

[19] Eurobarometer. Attitudes of European citizens towards the environment. Special Eurobarometer 501. 2020. Brussels: European Commission.

[20] Woo SL, Pepe O. Microbial consortia: promising probiotics as plant biostimulants for sustainable agriculture. Frontiers in plant science. 2018 Dec 4;9:1801. doi: 10.3389/fpls.2018.01801 30564264 PMC6288764

[21] Singh BK, Trivedi P, Egidi E, Macdonald CA, Delgado-Baquerizo M. Crop microbiome and sustainable agriculture. Nature Reviews Microbiology. 2020 Nov;18(11):601–2. doi: 10.1038/s41579-020-00446-y 33037425

[22] D’Hondt K, Kostic T, McDowell R, Eudes F, Singh BK, Sarkar S, et al. Microbiome innovations for a sustainable future. Nature Microbiology. 2021 Feb;6(2):138–42. doi: 10.1038/s41564-020-00857-w 33510435

[23] Ahmad M, Pataczek L, Hilger TH, Zahir ZA, Hussain A, Rasche F, et al. Perspectives of microbial inoculation for sustainable development and environmental management. Frontiers in microbiology. 2018 Dec 5;9:2992. doi: 10.3389/fmicb.2018.02992 30568644 PMC6289982

[24] Berg G, Köberl M, Rybakova D, Müller H, Grosch R, Smalla K. Plant microbial diversity is suggested as the key to future biocontrol and health trends. FEMS microbiology ecology. 2017 May 1;93(5). doi: 10.1093/femsec/fix050 28430944

[25] Menendez E, Garcia-Fraile P. Plant probiotic bacteria: solutions to feed the world. AIMS microbiology. 2017;3(3):502. doi: 10.3934/microbiol.2017.3.502 31294173 PMC6604988

[26] Khan MS, Zaidi A, Wani PA, Oves M. Role of plant growth promoting rhizobacteria in the remediation of metal contaminated soils. Environmental chemistry letters. 2009 Feb;7(1):1–9.

[27] Jiménez-Gómez A, Celador-Lera L, Fradejas-Bayón M, Rivas R. Plant probiotic bacteria enhance the quality of fruit and horticultural crops. AIMS microbiology. 2017;3(3):483. doi: 10.3934/microbiol.2017.3.483 31294172 PMC6604990

[28] Singh BK, Trivedi P. Microbiome and the future for food and nutrient security. Microbial biotechnology. 2017 Jan;10(1):50–3. doi: 10.1111/1751-7915.12592 28074557 PMC5270726

[29] Khalid M, Hassani D, Bilal M, Asad F, Huang D. Influence of bio-fertilizer containing beneficial fungi and rhizospheric bacteria on health promoting compounds and antioxidant activity of Spinacia oleracea L. Botanical studies. 2017 Dec;58(1):1–9. doi: 10.1186/s40529-016-0155-5 28815474 PMC5559411

[30] Hamouz K, Lachman J, Pivec V, Vokal B. Influence of environmental conditions and way of cultivation on the polyphenol and ascorbic acid content in potato tubers. Hortic Sci. 1999; 45:293–298.

[31] Rodríguez-Morató J, Xicota L, Fitó M, Farré M, Dierssen M, De la Torre R. Potential role of olive oil phenolic compounds in the prevention of neurodegenerative diseases. Molecules. 2015 Mar;20(3):4655–80. doi: 10.3390/molecules20034655 25781069 PMC6272603

[32] Mitchell AE, Hong YJ, Koh E, Barrett DM, Bryant DE, Denison RF, et al. Ten-year comparison of the influence of organic and conventional crop management practices on the content of flavonoids in tomatoes. Journal of agricultural and food chemistry. 2007 Jul 25;55(15):6154–9. doi: 10.1021/jf070344+ 17590007

[33] Ortuno A, Benavente-Garcia O, Castillo J, Alcaraz M, Vicente V, Del Rio JA. Beneficial action of Citrus flavonoids on multiple cancer-related biological pathways. Current cancer drug targets. 2007 Dec 1;7(8):795–809. doi: 10.2174/156800907783220435 18220529

[34] Alarcón-Flores MI, Romero-González R, Vidal JL, Frenich AG. Determination of phenolic compounds in artichoke, garlic and spinach by ultra-high-performance liquid chromatography coupled to tandem mass spectrometry. Food analytical methods. 2014 Nov;7(10):2095–106.

[35] Blum WE, Zechmeister-Boltenstern S, Keiblinger KM. Does soil contribute to the human gut microbiome?. Microorganisms. 2019 Sep;7(9):287. doi: 10.3390/microorganisms7090287 31450753 PMC6780873

[36] McFadden D. The choice theory approach to market research. Marketing science. 1986 Nov;5(4):275–97.

[37] Cranfield JA, Magnusson E. Canadian consumer’s willingness-to-pay for pesticide free food products: an ordered probit analysis. International Food and Agribusiness Management Review. 2003;6(1030-2016-82568).

[38] Kim J, Rasouli S, Timmermans H. Expanding scope of hybrid choice models allowing for mixture of social influences and latent attitudes: Application to intended purchase of electric cars. Transportation research part A: policy and practice. 2014 Nov 1;69:71–85.

[39] Hess S, Stathopoulos A. Linking response quality to survey engagement: a combined random scale and latent variable approach. Journal of Choice Modelling. 2013 Jun 1;7:1–2.

[40] Ben-Akiva M, Walker J, Bernardino AT, Gopinath DA, Morikawa T, Polydoropoulou A. Integration of choice and latent variable models. Perpetual motion: Travel behaviour research opportunities and application challenges. 2002 May:431–70.

[41] Hess S, Beharry-Borg N. Accounting for latent attitudes in willingness-to-pay studies: the case of coastal water quality improvements in Tobago. Environmental and Resource Economics. 2012 May;52(1):109–31.

[42] Daly A, Hess S, Patruni B, Potoglou D, Rohr C. Using ordered attitudinal indicators in a latent variable choice model: a study of the impact of security on rail travel behaviour. Transportation. 2012 Mar;39(2):267–97.

[43] Anderson JC, Gerbing DW. Structural equation modeling in practice: A review and recommended two-step approach. Psychological bulletin. 1988 May;103(3):411.

[44] Sok J, van der Lans IA, Hogeveen H, Elbers AR, Oude Lansink AG. Farmers’ preferences for bluetongue vaccination scheme attributes: an integrated choice and latent variable approach. Journal of Agricultural Economics. 2018 Jun;69(2):537–60.

[45] Bagozzi RP, Yi Y. Specification, evaluation, and interpretation of structural equation models. Journal of the academy of marketing science. 2012 Jan 1;40(1):8–34.

[46] Diamantopoulos A, Winklhofer HM. Index construction with formative indicators: An alternative to scale development. Journal of marketing research. 2001 May;38(2):269–77.

[47] Maher J, Pihlanto A, Kloos A. Sustainable Microbes in Agriculture- easily explained for farmers. Digital or Visual Products, Wageningen University & Research. 2020. Retrieved on 04 September 2020 from https://edepot.wur.nl/522592.

[48] Baker GA. Consumer preferences for food safety attributes in fresh apples: Market segments, consumer characteristics, and marketing opportunities. Journal of Agricultural and Resource Economics. 1999 Jul 1:80–97.

[49] Buzby JC, Fox JA, Ready RC, Crutchfield SR. Measuring consumer benefits of food safety risk reductions. Journal of Agricultural and Applied Economics. 1998;30(1379-2016-113415):69–82.

[50] Buzby JC, Ready RC, Skees JR. Contingent valuation in food policy analysis: a case study of a pesticide-residue risk reduction. Journal of Agricultural and Applied Economics. 1995;27(1379-2016-113234):613–25.

[51] Florax RJ, Travisi CM, Nijkamp P. A meta-analysis of the willingness to pay for reductions in pesticide risk exposure. European Review of Agricultural Economics. 2005 Dec 1;32(4):441–67.

[52] Bernard JC, Bernard DJ. Comparing parts with the whole: Willingness to pay for pesticide-free, non-GM, and organic potatoes and sweet corn. Journal of agricultural and resource economics. 2010 Dec 1:457–75.

[53] Katt F, Meixner O. A systematic review of drivers influencing consumer willingness to pay for organic food. Trends in Food Science & Technology. 2020 Apr 28.

[54] Yu X, Gao Z, Zeng Y. Willingness to pay for the “Green Food” in China. Food policy. 2014 Apr 1;45:80–7.

[55] Royne MB, Levy M, Martinez J. The public health implications of consumers’ environmental concern and their willingness to pay for an eco-friendly product. Journal of Consumer Affairs. 2011 Jun;45(2):329–43.

[56] Meixner O, Katt F. Assessing the Impact of COVID-19 on Consumer Food Safety Perceptions—A Choice-Based Willingness to Pay Study. Sustainability. 2020 Jan;12(18):7270.

[57] Blumenschein K, Johannesson M, Blomquist GC, Liljas B, O’Conor RM. Experimental results on expressed certainty and hypothetical bias in contingent valuation. Southern Economic Journal. 1998 Jul 1:169–77.

[58] Bechthold A., Boeing H., Schwedhelm C., Hoffmann G., Knüppel S., Iqbal K., et al. (2019). Food groups and risk of coronary heart disease, stroke and heart failure: A systematic review and dose-response meta-analysis of prospective studies. Critical Reviews in Food Science and Nutrition, 59(7), 1071–1090. 10.1080/10408398.2017.1392288 29039970

[59] Reipurth MF, Hørby L, Gregersen CG, Bonke A, Cueto FJ. Barriers and facilitators towards adopting a more plant-based diet in a sample of Danish consumers. Food quality and preference. 2019 Apr 1;73:288–92.

[60] Zizzo DJ, Parravano M, Nakamura R, Forwood S, Suhrcke M. The impact of taxation and signposting on diet: an online field study with breakfast cereals and soft drinks. Experimental Economics. 2021 Jan 29:1–31.

