# Supplementary material for: Consumer willingness to pay for plant-based foods produced using microbial applications to replace synthetic chemical inputs
Source: PLoS One. 2021 Dec 7;16(12):e0260488. doi: 10.1371/journal.pone.0260488 (PMC8651115; doi:10.1371/journal.pone.0260488)
Supplement: S1 File — (DOCX) [file pone.0260488.s001.docx]

**Consumer willingness to pay for plant-based foods produced using microbial applications to replace synthetic chemical inputs**

# Supplementary Material

**Supplementary Material A**: Frequency tables of respondents’ willingness-to-pay (WTP) by gender, age and level of education

**S1 Table**. Frequency tables of respondents’ WTP by gender (percentage of female (F) and male (M) respondents)

|  | *WTP_20%* ^a^ | | | *WTP_50%* ^a^ | | | *WTP_80%* ^a^ | | | *WTP_100%* ^a^ | | |
| --- | --- | --- | --- | --- | --- | --- | --- | --- | --- | --- | --- | --- |
| WTP | F | M | All | F | M | All | F | M | All | F | M | All |
| 0 | 11 | 18 | 13 | 6 | 9 | 7 | 4 | 8 | 5 | 1 | 5 | 3 |
| 1-10 euro cents | 26 | 29 | 27 | 18 | 23 | 19 | 13 | 15 | 14 | 11 | 11 | 11 |
| 11-20 euro cents | 32 | 23 | 29 | 33 | 29 | 32 | 18 | 23 | 20 | 12 | 14 | 13 |
| 21-50 euro cents | 19 | 18 | 19 | 27 | 22 | 25 | 36 | 25 | 32 | 22 | 29 | 25 |
| >50 euro cents | 12 | 13 | 13 | 16 | 18 | 16 | 28 | 29 | 29 | 53 | 41 | 49 |
| Total | 100 | 100 | 100 | 100 | 100 | 100 | 100 | 100 | 100 | 100 | 100 | 100 |

^a^ *WTP_20%*, *WTP_50%*, *WTP_80%* and *WTP_100%* refer to respondents’ WTP a premium for 1 kg of food product (i.e. consumer potato/wheat bread/tomato sauce) that is produced with a 20%, 50%, 80% and 100% less synthetical chemical use in primary production by replacement with microbial applications, respectively.

**S2 Table**. Frequency tables of respondents’ WTP by age

| WTP | Younger than 37 years | | Older than 37 years | | Overall (all age group) | |
| --- | --- | --- | --- | --- | --- | --- |
| *WTP_20% ^a^* | Frequency | Percent | Frequency | Percent | Frequency | Percent |
| 0 | 20 | 14 | 9 | 9 | 29 | 12 |
| 1-10 euro cents | 39 | 28 | 26 | 27 | 65 | 27 |
| 11-20 euro cents | 42 | 30 | 27 | 28 | 69 | 29 |
| 21-50 euro cents | 25 | 18 | 21 | 21 | 46 | 19 |
| >50 euro cents | 15 | 11 | 15 | 15 | 30 | 13 |
| Total | 141 | 100 | 98 | 100 | 239 | 100 |
| *WTP_50% ^a^* |  |  |  |  |  |  |
| 0 | 11 | 8 | 6 | 6 | 17 | 7 |
| 1-10 euro cents | 30 | 21 | 16 | 17 | 46 | 19 |
| 11-20 euro cents | 40 | 29 | 33 | 34 | 73 | 31 |
| 21-50 euro cents | 38 | 27 | 23 | 24 | 61 | 26 |
| >50 euro cents | 21 | 15 | 18 | 19 | 39 | 17 |
| Total | 140 | 100 | 96 | 100 | 236 | 100 |
| *WTP_80% ^a^* |  |  |  |  |  |  |
| 0 | 7 | 5 | 6 | 6 | 13 | 5 |
| 1-10 euro cents | 24 | 17 | 11 | 11 | 35 | 15 |
| 11-20 euro cents | 28 | 20 | 15 | 15 | 43 | 18 |
| 21-50 euro cents | 43 | 31 | 35 | 36 | 78 | 33 |
| >50 euro cents | 38 | 27 | 30 | 31 | 68 | 29 |
| Total | 140 | 100 | 97 | 100 | 237 | 100 |
| *WTP_100% ^a^* |  |  |  |  |  |  |
| 0 | 4 | 3 | 5 | 5 | 9 | 4 |
| 1-10 euro cents | 14 | 10 | 11 | 11 | 25 | 11 |
| 11-20 euro cents | 20 | 14 | 10 | 10 | 30 | 13 |
| 21-50 euro cents | 36 | 26 | 25 | 26 | 61 | 26 |
| >50 euro cents | 67 | 48 | 46 | 47 | 113 | 47 |
| Total | 141 | 100 | 97 | 100 | 238 | 100 |

^a^ *WTP_20%*, *WTP_50%*, *WTP_80%* and *WTP_100%* refer to respondents’ WTP a premium for 1 kg of food product (i.e. consumer potato/wheat bread/tomato sauce) that is produced with a 20%, 50%, 80% and 100% less synthetical chemical use in primary production by replacement with microbial applications, respectively.

**S3 Table**. Frequency tables of respondents’ WTP by level of education (percentage of respondents without and with higher education)

|  | *WTP_20%* ^a^ | | | *WTP_50%* ^a^ | | | *WTP_80%* ^a^ | | | *WTP_100%* ^a^ | | |
| --- | --- | --- | --- | --- | --- | --- | --- | --- | --- | --- | --- | --- |
| WTP | Without | With | All | Without | With | All | Without | With | All | Without | With | All |
| 0 | 16 | 13 | 13 | 9 | 7 | 8 | 7 | 6 | 6 | 4 | 3 | 4 |
| 1-10 euro cents | 18 | 29 | 27 | 16 | 20 | 19 | 9 | 15 | 14 | 9 | 12 | 11 |
| 11-20 euro cents | 36 | 27 | 28 | 27 | 32 | 31 | 16 | 19 | 18 | 9 | 14 | 13 |
| 21-50 euro cents | 16 | 19 | 19 | 31 | 24 | 25 | 31 | 33 | 33 | 18 | 27 | 25 |
| >50 euro cents | 16 | 13 | 13 | 18 | 17 | 17 | 38 | 26 | 29 | 60 | 45 | 47 |
| Total | 100 | 100 | 100 | 100 | 100 | 100 | 100 | 100 | 100 | 100 | 100 | 100 |

^a^ *WTP_20%*, *WTP_50%*, *WTP_80%* and *WTP_100%* refer to respondents’ WTP a premium for 1 kg of food product (i.e. consumer potato/wheat bread/tomato sauce) that is produced with a 20%, 50%, 80% and 100% less synthetical chemical use in primary production by replacement with microbial applications, respectively.

**Supplementary Material B**: Estimation results of the full latent variable model

**S4 Table**. Estimation results of the latent variable model by including all the eleven indicators in the measurement model ^a^

|  | Promotion oriented | | Prevention oriented | |
| --- | --- | --- | --- | --- |
| *Structural model* | Coefficient | Std. Err. | Coefficient | Std. Err. |
| Household size | 0.15* | 0.08 | -0.16* | 0.09 |
| Age | 0.15** | 0.07 |  |  |
| Higher education |  |  | -0.15** | 0.07 |
| Gender | -0.11 | 0.08 | 0.13 | 0.08 |
| Residence | 0.13* | 0.08 | -0.18** | 0.08 |
| Income | -0.12* | 0.07 |  |  |
| Expenditure |  |  | 0.20*** | 0.07 |
| Consumption frequency |  |  | 0.14* | 0.08 |
| Product type | 0.18*** | 0.08 | 0.18* | 0.09 |
| Purchasing place | 0.10 | 0.07 |  |  |
| Potato |  |  | 0.15* | 0.08 |
| Germany | -0.21*** | 0.09 | 0.13 | 0.10 |
| Netherlands | -0.13* | 0.08 | 0.21** | 0.09 |
| Other country |  |  | 0.11 | 0.08 |
| Environmental concern |  |  | -0.23*** | 0.07 |
| Health concern | 0.30*** | 0.07 |  |  |
| Attitude towards microbial application | 0.17** | 0.08 | -0.17** | 0.09 |
| *Measurement model* |  |  |  |  |
| FCM1 | 0.45*** | 0.07 |  |  |
| FCM2 | 0.71*** | 0.05 |  |  |
| FCM3 | 0.06 | 0.09 |  |  |
| FCM4 | 0.51*** | 0.07 |  |  |
| FCM5 | 0.84*** | 0.04 |  |  |
| FCM6 | 0.43*** | 0.07 |  |  |
| FCM7 |  |  | 0.30*** | 0.08 |
| FCM8 |  |  | 0.83*** | 0.05 |
| FCM9 |  |  | -0.02 | 0.09 |
| FCM10 |  |  | 0.07 | 0.09 |
| FCM11 |  |  | 0.74*** | 0.06 |
| *Goodness-of-fit measures* |  |  |  |  |
| RMSEA | 0.07 ^b^ |  |  |  |
| CFI | 0.65 ^b^ |  |  |  |
| SRMR | **0.07** ^b^ |  |  |  |
| *Error term covariances* |  |  |  |  |
| Promotion oriented | 0.57 | 0.07 |  |  |
| Prevention oriented | -0.70*** | 0.09 | 0.73 | 0.07 |

^a^ *N* = 158. ^b^ The cut-off values for acceptance of the goodness-of-fit of the specified model are <= 0.06 for RMSEA, >=0.95 for CFI and <=0.08 for SRMR. The SRMR measure of model goodness-of-fit indicates that the indicators used in the latent variables’ construction are acceptable in defining the constructs.

Likelihood ratio test of model vs. saturated: Chi^2^(261) = 464***.

***, **, *Significant at 1%, 5% and 10% critical levels, respectively.

**Supplementary Material C**: Ordered logistic estimation results of the WTP regression model

The maximum likelihood estimation results and the respective marginal effects of the ordered logistic WTP model are presented in S5 and S6 Tables. The likelihood ratio test shows that the included explanatory variables are jointly significant in explaining the variation in predicted probabilities of WTP at 1% critical level (S5 Table). Both the promotion- and prevention-oriented FCM latent variables do not have a significant association with the predicted probabilities of the WTP categories, except for the case of *WTP_80%* where the promotion-oriented construct is statistically significant (S5 Table). The only significant explanatory variables that affect the predicted probabilities of WTP are environmental and health concerns, and the dummy variables used to codify potato, Germany, Finland and other countries. The average predicted probability of the WTP categories for a 20% reduction in chemical use is lower by about 3% and 5% for a one unit increase in environmental and health concern, respectively (S6 Table). This implies that highly (environmental and health) concerned consumers are less likely to pay premiums for food products that are produced with microbial applications.

**S5 Table. Maximum likelihood estimation results of the ordered logistic WTP model**

|  | *WTP_20%* | | *WTP_50%* | | *WTP_80%* | | *WTP_100%* | |
| --- | --- | --- | --- | --- | --- | --- | --- | --- |
| Variables | Coef. | SE | Coef. | SE | Coef. | SE | Coef. | SE |
| Promotion oriented FCM | 0.59 | 0.50 | 0.51 | 0.54 | 1.05* | 0.60 | 0.07 | 0.64 |
| Prevention oriented FCM | 0.25 | 0.26 | 0.15 | 0.28 | 0.43 | 0.32 | -0.24 | 0.30 |
| Attitude | -0.06 | 0.22 | 0.15 | 0.20 | 0.33* | 0.19 | 0.35* | 0.19 |
| Microbial knowledge | 0.09 | 0.14 | 0.09 | 0.13 | 0.06 | 0.13 | -0.08 | 0.14 |
| Microbial health risk | -0.15 | 0.25 | -0.31 | 0.25 | -0.20 | 0.23 | -0.28 | 0.22 |
| Environmental concern | 0.35* | 0.21 | 0.55** | 0.20 | 0.29* | 0.17 | 0.40** | 0.17 |
| Health concern | 0.49** | 0.20 | 0.39* | 0.21 | 0.47** | 0.21 | 0.68*** | 0.24 |
| Household size | 0.10 | 0.11 | 0.10 | 0.10 | 0.07 | 0.12 | 0.01 | 0.12 |
| Age | -0.01 | 0.01 | -0.02* | 0.01 | -0.02* | 0.01 | -0.02** | 0.01 |
| Higher education | 0.22 | 0.33 | 0.23 | 0.30 | -0.10 | 0.32 | -0.47 | 0.41 |
| Gender | 0.11 | 0.30 | 0.25 | 0.31 | 0.08 | 0.33 | -0.20 | 0.31 |
| Residence | -0.15 | 0.29 | -0.18 | 0.28 | -0.07 | 0.30 | -0.10 | 0.33 |
| Income | 0.02 | 0.08 | 0.05 | 0.08 | 0.04 | 0.08 | 0.09 | 0.08 |
| Expenditure | 0.04 | 0.15 | 0.03 | 0.17 | -0.04 | 0.16 | 0.05 | 0.17 |
| Consumption frequency | 0.25 | 0.19 | 0.25 | 0.17 | 0.32** | 0.17 | 0.32** | 0.15 |
| Product type | -0.36 | 0.35 | -0.07 | 0.34 | -0.39 | 0.38 | -0.04 | 0.37 |
| Purchasing place | 0.05 | 0.21 | 0.08 | 0.16 | 0.12 | 0.17 | 0.17 | 0.18 |
| Potato | 1.76** | 0.73 | 1.17* | 0.65 | 1.11* | 0.64 | 0.63 | 0.61 |
| Tomato | -0.06 | 0.41 | -0.02 | 0.40 | 0.35 | 0.40 | 0.04 | 0.40 |
| Germany | 1.76*** | 0.55 | 1.59*** | 0.54 | 1.40*** | 0.53 | 0.88* | 0.51 |
| Netherlands | -0.18 | 0.45 | 0.13 | 0.51 | 0.02 | 0.52 | 0.04 | 0.53 |
| Finland | 1.63*** | 0.60 | 1.80* | 1.00 | 0.69 | 0.81 | 0.65 | 0.77 |
| Other country | -1.11** | 0.55 | -0.91* | 0.51 | -1.10** | 0.54 | -0.75 | 0.55 |
| Threshold 1 | 3.19 | 1.62 | 2.97 | 1.69 | 2.91 | 1.49 | 2.29 | 1.70 |
| Threshold 2 | 4.98 | 1.61 | 4.88 | 1.72 | 4.73 | 1.47 | 4.05 | 1.68 |
| Threshold 3 | 6.50 | 1.63 | 6.68 | 1.75 | 5.95 | 1.48 | 5.08 | 1.68 |
| Threshold 4 | 7.95 | 1.67 | 8.33 | 1.77 | 7.68 | 1.51 | 6.59 | 1.72 |
| *Goodness-of-fit* |  |  |  |  |  |  |  |  |
| Observations | 213 | | 211 | | 211 | | 213 | |
| Log likelihood | -299.29 | | -283.12 | | -279.05 | | -242.43 | |
| Likelihood ratio test of zero slope coefficients | 59.36*** | | 61.47*** | | 59.43*** | | 69.43*** | |
| Pseudo R^2^ | 0.09 | | 0.10 | | 0.09 | | 0.12 | |

Abbreviations: *WTP_20%*, *WTP_50%*, *WTP_80%* and *WTP_100%* refer to respondents’ willingness to pay a premium for 1 kg of food product (i.e. consumer potato/wheat bread/tomato sauce) that is produced with a 20%, 50%, 80% and 100% less chemical use in farming due to microbial applications, respectively.

***, **, *Significant at 1%, 5% and 10% critical levels, respectively.

**S6 Table. Marginal effects of the ordered logistic regression estimation of the WTP model**

|  | *WTP_20%* | | *WTP_50%* | | *WTP_80%* | | *WTP_100%* | | |
| --- | --- | --- | --- | --- | --- | --- | --- | --- | --- |
| Variables | Coef. | SE | Coef. | SE | Coef. | SE | Coef. | SE |  |
| Promotion oriented FCM | -0.056 | 0.049 | -0.026 | 0.029 | -0.039 | 0.027 | -0.002 | 0.017 |  |
| Prevention oriented FCM | -0.024 | 0.026 | -0.008 | 0.015 | -0.016 | 0.014 | 0.006 | 0.008 |  |
| Attitude | 0.006 | 0.020 | -0.008 | 0.011 | -0.012 | 0.008 | -0.009 | 0.007 |  |
| Microbial knowledge | -0.009 | 0.013 | -0.005 | 0.006 | -0.002 | 0.005 | 0.002 | 0.004 |  |
| Microbial health risk | 0.014 | 0.024 | 0.015 | 0.013 | 0.007 | 0.009 | 0.007 | 0.006 |  |
| Environmental concern | -0.033* | 0.019 | -0.028** | 0.012 | -0.011* | 0.007 | -0.010* | 0.006 |  |
| Health concern | -0.047** | 0.020 | -0.020* | 0.012 | -0.017** | 0.009 | -0.018** | 0.009 |  |
| Household size | -0.010 | 0.010 | -0.005 | 0.005 | -0.003 | 0.005 | 0.000 | 0.003 |  |
| Age | 0.000 | 0.001 | 0.001* | 0.001 | 0.001 | 0.000 | 0.001 | 0.000 |  |
| Higher education | -0.021 | 0.032 | -0.012 | 0.015 | 0.004 | 0.012 | 0.012 | 0.012 |  |
| Gender | -0.010 | 0.029 | -0.013 | 0.016 | -0.003 | 0.012 | 0.005 | 0.009 |  |
| Residence | 0.014 | 0.028 | 0.009 | 0.015 | 0.002 | 0.011 | 0.003 | 0.009 |  |
| Income | -0.001 | 0.008 | -0.002 | 0.004 | -0.002 | 0.003 | -0.002 | 0.002 |  |
| Expenditure | -0.004 | 0.014 | -0.001 | 0.008 | 0.001 | 0.006 | -0.001 | 0.005 |  |
| Consumption frequency | -0.023 | 0.019 | -0.013 | 0.009 | -0.012* | 0.007 | -0.008* | 0.005 |  |
| Product type | 0.034 | 0.034 | 0.004 | 0.017 | 0.015 | 0.015 | 0.001 | 0.010 |  |
| Purchasing place | -0.005 | 0.019 | -0.004 | 0.008 | -0.004 | 0.007 | -0.004 | 0.005 |  |
| Potato | -0.167** | 0.071 | -0.059* | 0.036 | -0.041 | 0.027 | -0.016 | 0.017 |  |
| Tomato | 0.006 | 0.038 | 0.001 | 0.020 | -0.013 | 0.016 | -0.001 | 0.010 |  |
| Germany | -0.167*** | 0.054 | -0.080*** | 0.032 | -0.052** | 0.026 | -0.023 | 0.016 |  |
| Netherlands | 0.017 | 0.043 | -0.007 | 0.025 | -0.001 | 0.019 | -0.001 | 0.014 |  |
| Finland | -0.154*** | 0.059 | -0.091* | 0.055 | -0.026 | 0.031 | -0.017 | 0.021 |  |
| Other country | 0.105** | 0.051 | 0.046* | 0.027 | 0.041* | 0.024 | 0.020 | 0.015 |  |

Abbreviations: *WTP_20%*, *WTP_50%*, *WTP_80%* and *WTP_100%* refer to respondents’ willingness to pay a premium for 1 kg of food product (i.e. consumer potato/wheat bread/tomato sauce) that is produced with a 20%, 50%, 80% and 100% less chemical use in farming due to microbial applications, respectively.

***, **, *Significant at 1%, 5% and 10% critical levels, respectively.
